# A High-Temperature Semi-Coupled Damage Model for 7075 Aluminum Alloy Considering Temperature and Strain Rate Effects

**DOI:** 10.3390/ma19214650

**Published:** 2026-10-31

**Authors:** Dan Yao, Muyu Li, Zhiyong Chen, Yixu Niu, Bin Li, Dalei Zu, Yingping Guan

**Affiliations:** 1 School of Intelligent Manufacturing, Luoyang Institute of Science and Technology, Luoyang 471023, China; yaodanysu@163.com (D.Y.); niuniuyixu@163.com (Y.N.); libin@lit.edu.cn (B.L.); zudalei@163.com (D.Z.); 2 Henan Key Laboratory of Green Building Materials Manufacturing and Intelligent Equipment, Luoyang 471023, China; 3 School of Intelligent Vehicle Engineering, Luoyang Institute of Science and Technology, Luoyang 471023, China; 1854@163.com; 4 School of Mechanical Engineering, Yanshan University, Qinhuangdao 066004, China; gyp@ysu.edu.cn

**Keywords:** 7075 aluminum alloy, semi-coupled fracture criterion, high-temperature damage model, damage evolution

## Abstract

Fracture is a common and fatal defect in the hot stamping of aluminum alloys. Accurate prediction of high-temperature fracture behavior is crucial for forming process planning. However, existing high-temperature fracture criteria suffer from a trade-off between predictive capability and parameter calibration difficulty, which limits their industrial application. This study establishes a high-temperature semi-coupled damage model by incorporating strain rate and temperature terms into a semi-coupled fracture framework and coupling it with a viscoplastic constitutive model. This model maintains the convenience of parameter calibration while describing the nonlinear characteristics of damage evolution using a hyperbolic sine function. The model was applied to the finite element analysis of 7075 aluminum alloy. For hot tensile tests, the simulated load–displacement responses, fracture displacements, and strain fields agree with the experiments, with a maximum load–displacement root mean square error (RMSE) of 0.047 kN and a maximum fracture displacement relative error of 3.141%. For isothermal bulging validation tests, the average relative error for fracture location prediction is 5.10%, and the maximum relative error for normalized thickness is 6.67%. These results demonstrate that within 350–450 °C and 0.001–0.1 s^−1^, the proposed model accurately predicts the damage initiation, evolution, and fracture behavior.

## 1. Introduction

Owing to their low density, high specific strength, and good corrosion resistance, aluminum alloys are regarded as ideal lightweight materials to replace conventional steel and have been widely used in the automotive, aerospace, and other fields [1,2]. However, aluminum alloys exhibit poor formability at room temperature and are prone to quality issues such as fracture, wrinkling, and springback, thus typically requiring hot stamping. During hot stamping, the microstructure undergoes significant changes under the coupled effects of strain rate and temperature, leading to the degradation of macroscopic mechanical properties and ultimately inducing fracture, which affects the quality of the formed parts [3,4,5]. Consequently, accurate prediction of the high-temperature fracture behavior of aluminum alloys is of great significance for forming process planning, improving production efficiency, and reducing manufacturing costs.

Compared with other fracture prediction methods for metals, fracture criteria offer higher flexibility and broader applicability. Various ductile fracture criteria have been proposed for metallic materials. Based on the interaction between damage and plastic behavior, these fracture criteria can be classified into two categories: coupled and uncoupled fracture criteria. Uncoupled fracture criteria neglect the influence of damage on plastic behavior and treat fracture as a subsequent state after plastic deformation develops to a certain stage, offering the advantages of a simple structural form and convenient parameter calibration [6,7,8]. To extend the uncoupled fracture criterion to high-temperature conditions, researchers have proposed various modified models by introducing temperature- and strain rate-dependent terms. Du et al. [9] introduced a strain rate and a temperature scaling factor into the MMC fracture criterion, and established an extended model applicable for predicting the high-temperature fracture behavior of AA5383 alloy. Valoppi et al. [10] considered the influence of lode angle and deformation temperature on fracture behavior, and incorporated polynomial expressions related to temperature into the Johnson–Cook fracture criterion. Huang et al. [11] embedded the Z parameter and the DRX volume fraction into the conventional ductile fracture criterion, and proposed an extended ductile fracture criterion that takes into account the influence of stress state and dynamic recrystallization. Kacem et al. [12] systematically investigated the effects of temperature and stress state on fracture behavior and introduced a linear temperature term into the Lou criterion, proposing an extended Lou ductile fracture criterion with high prediction accuracy. The aforementioned extended models have to some extent improved the prediction accuracy at high temperatures. However, all these models ignore the feedback effect of damage on the plastic behavior, making it difficult to accurately describe the damage-induced softening behavior of the material during high-temperature deformation.

Coupled fracture criteria consider that damage continuously evolves within the material during plastic deformation, and that the accumulation of damage leads to progressive degradation of material properties, ultimately causing fracture [13,14,15]. Compared to uncoupled criteria, such models can more accurately describe the high-temperature mechanical behavior of materials. For example, Imran et al. [16] and Shang et al. [17] considered the interaction between dynamic recrystallization and damage and established a GTN mesoscopic damage model for hot forming. Wen et al. [18] developed a constitutive model based on dislocation density that couples flow softening, phase transformation, and damage mechanisms to predict the hot tensile behavior, β phase fraction, and damage evolution of Ti6Al4V alloy. Tang et al. [19] considered the interaction among dislocation density, grain size, and the ductile fracture model and established a set of improved viscoplastic damage constitutive models, which can reveal the fracture mode and grain size evolution of 7075 alloy during hot stamping. Although the above studies adopted different damage description frameworks, they all suffer from complex structures and highly coupled parameters, making parameter solving difficult to a certain extent and limiting their widespread application.

Researchers have established a semi-coupled fracture criterion in recent years by clearly distinguishing between damage and fracture. This criterion activates the damage variable only during the softening stage, thereby describing the entire process from damage initiation to fracture [20,21,22]. Such criteria offer both high descriptive accuracy and convenient parameter calibration, showing broad application prospects. On this basis, Li et al. [23] combined an anisotropic model with the semi-coupled fracture criterion and used it to predict the fracture of DP1000 steel caused by anisotropy in hole expansion tests. Zeng et al. [24,25] established a rate- and temperature-dependent semi-coupled fracture criterion and applied it to predict the fracture behavior of H30 steel. However, current studies on semi-coupled fracture criteria are mainly limited to room temperature, and high-temperature semi-coupled fracture criteria remain scarce. The main challenge is that damage evolution at high temperatures exhibits significant nonlinear characteristics, and the strong coupling between strain rate and temperature makes the linear damage evolution assumption in room-temperature semi-coupled criteria invalid [26,27].

To establish a high-temperature fracture criterion that can accurately describe the entire process of aluminum alloys from damage initiation to fracture while maintaining simple parameter calibration, this study proposes a high-temperature semi-coupled damage model. Strain rate and temperature terms are introduced into damage initiation, damage evolution and fracture model and coupled with a high-temperature viscoplastic constitutive model. The model parameters are calibrated using an experimental–numerical hybrid method, and the proposed model is applied to finite element analysis of hot tensile and isothermal bulging tests of 7075 aluminum alloy to verify its predictive capability. This model maintains the convenience of parameter calibration while significantly improving the prediction accuracy of high-temperature fracture behavior.

## 2. Materials and Methods

### 2.1. Establishment of the High-Temperature Semi-Coupled Damage Model

#### 2.1.1. High-Temperature Viscoplastic Constitutive Model

During high-temperature deformation, the plastic strain rate of metals exhibits a highly nonlinear relationship with viscous stress, and viscoplastic flow occurs only after the initial yield strength is overcome. After plastic deformation begins, the rapid multiplication of dislocation density leads to work hardening, which increases the deformation resistance of the material. Therefore, the viscoplastic flow equation can be expressed as [28]:

(1) $${\dot{\varepsilon }}_{p}={\left(\frac{\sigma -H-k}{K}\right)}^{n_{1}}$$

where ${\dot{\varepsilon }}_{p}$ denotes the plastic strain rate; *H* is the hardening constant; σ is the equivalent stress; *k* and *K* denote the initial yield strength and the resistance coefficient, respectively, both of which are temperature-dependent; and *n*_1_ is the viscosity exponent.

The normalized dislocation density $\bar{\rho }$ is used to represent the relative dislocation density during high-temperature deformation, and the relationship between the hardening constant *H* and the normalized dislocation density $\bar{\rho }$ is expressed as [28]:

(2) $$H=B{\bar{\rho }}^{1/2}$$

where *B* is a temperature-dependent material constant.

During high-temperature deformation, the continuous multiplication and accumulation of dislocations lead to an increasing dislocation density. Simultaneously, the increased dislocation density elevates the stored energy in the metal, promoting dynamic recovery. This process annihilates dislocations, partially offsetting work hardening and thereby reducing the rate of flow stress increase. The dislocation density evolution equation is expressed as [29]:

(3) $$\dot{\bar{\rho }}=A\left(1-\bar{\rho }\right)\left|{\dot{\varepsilon }}_{p}\right|-C{\bar{\rho }}^{n_{2}}$$

where $\dot{\bar{\rho }}$ is the evolution rate of the normalized dislocation density; *n*_2_ is a temperature-independent material constant; *A* and *C* are temperature-dependent material constants. The first term describes the dislocation multiplication caused by plastic deformation and the dislocation annihilation caused by dynamic recovery, while the second term describes the static recovery process.

Considering the elastic stage, the equivalent stress is expressed as:

(4) $$\sigma =E\left(\varepsilon_{T}-\varepsilon_{p}\right)$$

where $\varepsilon_{T}$ and $\varepsilon_{p}$ are the total strain and equivalent plastic strain, respectively; *E* represents the elastic modulus.

In the above model, the Arrhenius equation is introduced to describe the effect of temperature. Therefore, the expressions for the temperature-dependent material constants are expressed as [29]:

(5) $$\left\{\begin{array}{l} A=A_{0}\exp(\frac{Q_{A}}{R_{g}T})\\ C=C_{0}\exp(-\frac{Q_{C}}{R_{g}T})\\ B=B_{0}\exp(\frac{Q_{B}}{R_{g}T})\\ K=K_{0}\exp(\frac{Q_{K}}{R_{g}T})\\ k=k_{0}\exp(\frac{Q_{k}}{R_{g}T})\\ E=E_{0}\exp(\frac{Q_{E}}{R_{g}T}) \end{array}\right.$$

where *Q_A_*, *Q_B_*, *Q_C_*, *Q_K_*, *Q_k_* and *Q_E_* denote the thermal activation energies; *A*_0_, *C*_0_, *B*_0_, *K*_0_, *k*_0_, and *E*_0_ are material constants; *R_g_* is the molar gas constant, with a value of 8.314 J/(mol·K); and *T* is the Kelvin temperature.

#### 2.1.2. High-Temperature Semi-Coupled Damage Model

Based on the room-temperature semi-coupled ductile fracture criterion [21], the high-temperature damage initiation, damage evolution, and fracture models were constructed. Since the mechanical properties of materials at high temperatures are strongly affected by temperature and strain rate, these effects must be incorporated into the damage initiation and fracture models. Previous studies have generally adopted two approaches to address this issue. The first approach defines the model parameters as functions dependent on temperature and strain rate, as seen in the coupled damage viscoplastic constitutive model [19]. The second approach introduces explicit strain rate and temperature terms into the original model, similar to the Johnson–Cook model [6]. Adopting the second approach, this study incorporates strain rate and temperature terms into the uncoupled ductile fracture criterion. The damage initiation model ${\bar{\varepsilon }}_{ddi}^{p}$ is expressed as:

(6) $${\bar{\varepsilon }}_{ddi}^{p}\left(\eta ,\bar{\theta }\right)=\frac{b_{3}}{{\left(\frac{2}{\sqrt{{\bar{\theta }}^{2}+3}}\right)}^{b_{1}}{\left(\frac{\langle 1+3\eta \rangle }{2}\right)}^{b_{2}}}\left[1+F_{1}\ln\left(\frac{\dot{\bar{\varepsilon }}}{{\dot{\bar{\varepsilon }}}^{*}}\right)\right]\left[1+F_{2}\left(\frac{T-T^{*}}{T_{m}-T^{*}}\right)\right]$$

(7) $$I_{dd}=\int_{0}^{{\bar{\varepsilon }}^{p}}\frac{d{\bar{\varepsilon }}^{p}}{{\bar{\varepsilon }}_{ddi}^{p}\left(\eta ,\bar{\theta }\right)}$$

where η and $\bar{\theta }$ are the stress triaxiality and Lode angle parameter; *b*_1_, *b*_2_ and *b*_3_ are material parameters characterizing damage; *F*_1_ and *F*_2_ are the parameters related to the strain rate and temperature influence, respectively; $\dot{\bar{\varepsilon }}$ and ${\dot{\bar{\varepsilon }}}^{*}$ are the strain rate and reference strain rate, respectively; *T* is the current temperature; $T^{*}$ is the reference temperature; $T_{m}$ is the melting point; and $I_{dd}$ is the damage initiation indicator.

Once $I_{dd}$ reaches 1, damage evolution is activated and the material enters the softening stage. Compared with room-temperature deformation, the damage mechanisms of aluminum alloys under high-temperature deformation are more complex, being related not only to the stress state but also affected by deformation temperature, strain rate, and deformation degree. Meanwhile, microstructure evolution also significantly influences the damage evolution behavior. The coupling effects of these factors lead to pronounced nonlinear characteristics in the damage evolution of aluminum alloys at high temperatures, and the rate of damage accumulation increases significantly with increasing deformation degree. Referring to the studies by Mohamed et al. [30] and Lin et al. [31], this study adopts a hyperbolic sine function to describe the damage evolution and introduces a plastic strain term to represent the nonlinear effect of deformation on damage accumulation. In addition, Johnson and Cook [6] showed that under tensile stress states, the stress triaxiality plays a dominant role, and the fracture strain decreases exponentially with increasing stress triaxiality. To reflect the effect of stress triaxiality on damage, a stress triaxiality term is introduced into the model. In summary, the high-temperature damage evolution model is expressed as:

(8) $$\dot{D}=\frac{{\bar{\sigma }}_{ddi}^{c}}{G_{f}}\sinh\left[m\left({\bar{\varepsilon }}^{p}-{\bar{\varepsilon }}_{ddi}^{p}\right)\right]\langle \eta \rangle \dot{\bar{\varepsilon }}$$

where $\dot{D}$ denotes the damage rate; ${\bar{\sigma }}_{ddi}^{c}$ is the equivalent stress at the damage initiation; *m* is a parameter reflecting the effect of plastic strain; and $G_{f}$ denotes the energy dissipated at a material point between the damage initiation and fracture.

Similarly, referring to the damage initiation model, the fracture model ${\bar{\varepsilon }}_{df}^{p}$ and fracture indicator $I_{df}$ under high temperatures are expressed as:

(9) $${\bar{\varepsilon }}_{df}^{p}\left(\eta ,\bar{\theta }\right)=\frac{c_{3}}{{\left(\frac{2}{\sqrt{{\bar{\theta }}^{2}+3}}\right)}^{c_{1}}{\left(\frac{\langle 1+3\eta \rangle }{2}\right)}^{c_{2}}}\left[1+F_{3}\ln\left(\frac{\dot{\bar{\varepsilon }}}{{\dot{\bar{\varepsilon }}}^{*}}\right)\right]\left[1+F_{4}\left(\frac{T-T^{*}}{T_{m}-T^{*}}\right)\right]$$

(10) $$I_{df}=\int_{{\bar{\varepsilon }}_{ddi}^{p,c}}^{{\bar{\varepsilon }}^{p}}\frac{d{\bar{\varepsilon }}^{p}}{{\bar{\varepsilon }}_{df}^{p}\left(\eta ,\bar{\theta }\right)-{\bar{\varepsilon }}_{ddi}^{p}\left(\eta ,\bar{\theta }\right)}$$

where *c*_1_, *c*_2_ and *c*_3_ are material parameters characterizing fracture; *F*_3_ and *F*_4_ are the parameters related to the strain rate and the temperature influence, respectively; ${\bar{\varepsilon }}_{ddi}^{p,c}$ is the critical equivalent plastic strain at damage initiation.

When $I_{df}$ reaches 1, the material completely loses its load-carrying capacity, resulting in final fracture.

Following the theoretical framework of the semi-coupled fracture criterion, the aforementioned damage initiation, damage evolution, and fracture models are incorporated into the high-temperature viscoplastic constitutive model to formulate a high-temperature semi-coupled damage model, which is expressed as:

(11) $$\left\{\begin{array}{l} {\dot{\varepsilon }}_{p}={\left(\frac{\sigma /(1-D)-H-k}{K}\right)}^{n_{1}}\\ H=B{\bar{\rho }}^{1/2}\\ \dot{\bar{\rho }}=A(1-\bar{\rho })\left|{\dot{\varepsilon }}_{p}\right|-C{\bar{\rho }}^{n_{2}}\\ \dot{D}=\frac{{\bar{\sigma }}_{ddi}^{c}}{G_{f}}\sinh\left[m\left(\varepsilon^{p}-\varepsilon_{ddi}^{p}\right)\right]\langle \eta \rangle \dot{\bar{\varepsilon }}\\ \sigma =E(1-D)(\varepsilon_{T}-\varepsilon_{p}) \end{array}\right.$$

The damage variable *D* is a piecewise function. When $I_{dd}$ is less than 1, damage has not yet initiated, and the damage variable is 0. When $I_{dd}$ reaches 1, damage begins to accumulate in a nonlinear manner. When $I_{df}$ reaches 1, it indicates that the damage has accumulated to a critical state, the material fractures regardless of the value of the damage variable. The piecewise function expression for the damage variable is given by [20]:

(12) $$D=\left\{\begin{array}{l} 0\;\;\;\;\;I_{dd}<1\\ D\;\;\;\;\;I_{dd}\geq 1\wedge I_{df}<1\\ 1\;\;\;\;\;I_{dd}\geq 1\wedge I_{df}\geq 1 \end{array}\right.$$

### 2.2. Experiments

To investigate the deformation behavior and fracture response of AA7075-T6 alloy across a range of temperatures and strain rates, three types of experiments were designed: uniaxial hot tensile tests, hot fracture tests, and isothermal bulging tests. The uniaxial hot tensile tests were used to obtain the high-temperature mechanical properties and to calibrate the viscoplastic constitutive model. The hot fracture tests including three notched tensile (NTR) specimens and one shear (SH) specimen were used to obtain the fracture response under different deformation conditions and stress states, and to calibrate the damage initiation, damage evolution, and fracture models. The isothermal bulging tests were completely independent of the calibration process and were used exclusively to validate the predictive capability of the proposed model. The experimental design and purposes are summarized in Table 1.

#### 2.2.1. Hot Tensile Test

**Figure 1:**
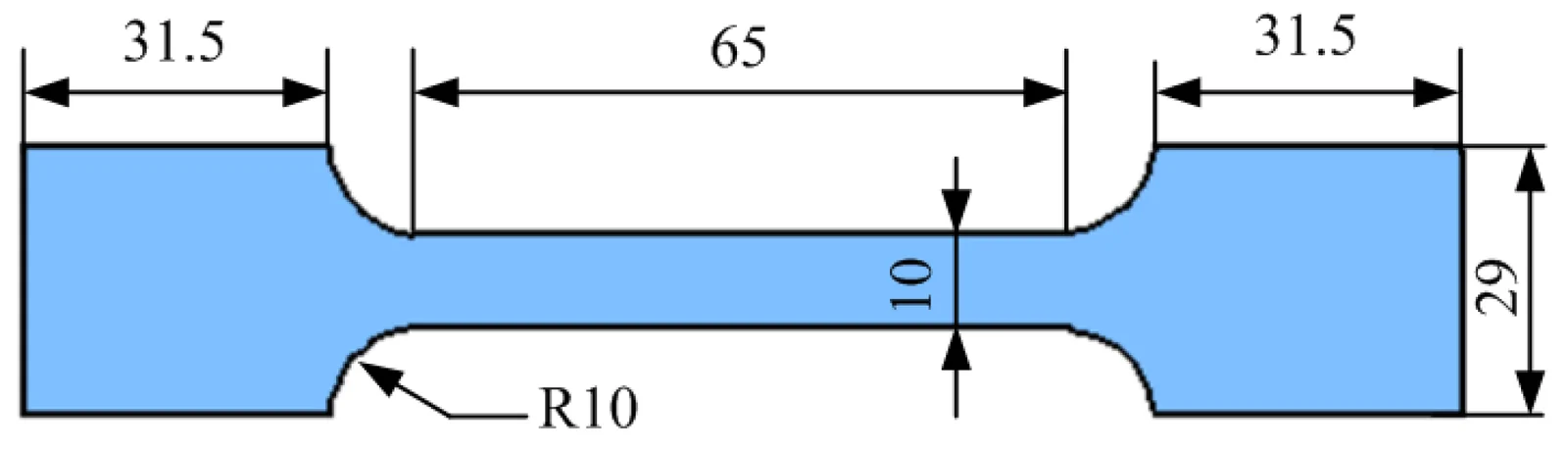
Geometric dimensions of the specimen in hot uniaxial tension test (mm).

**Figure 2:**
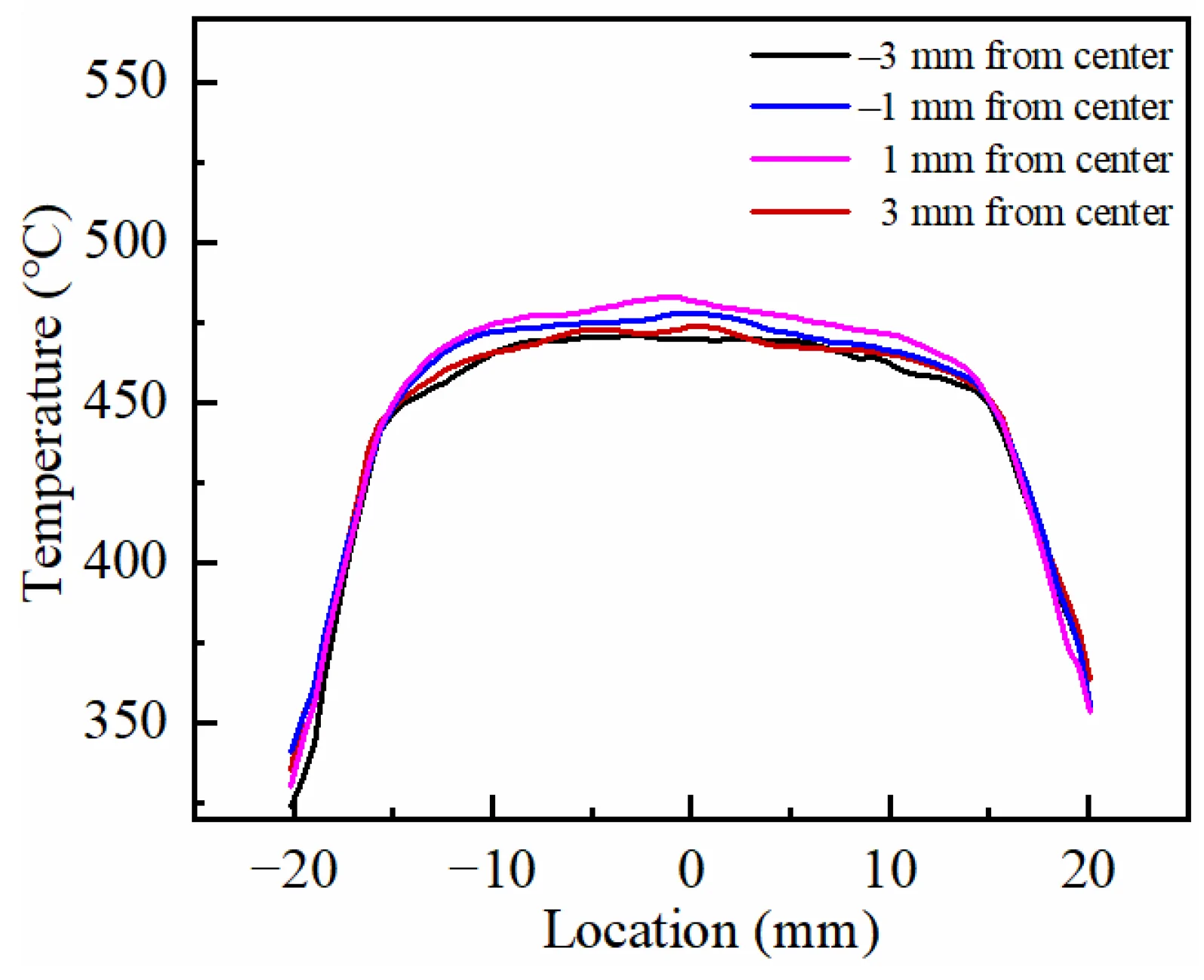
Temperature field along the gauge length of the specimen.

The material used in this study was 2 mm thick 7075-T6 aluminum alloy sheet. To obtain the mechanical properties at high temperatures, a hot tensile specimen was designed according to the international standard ISO 6892-2:2011 [32], and dimensions are shown in Figure 1. All hot tensile tests were conducted on a 10 kN hydraulic universal testing machine equipped with an induction heating system. To determine the extent of the uniform temperature zone during the hot tensile test, the surface temperature field of the specimen was acquired using an infrared thermal imager (FLIR T460, FLIR Systems, Wilsonville, OR, USA) prior to testing. Four longitudinal temperature distribution curves were extracted at equal intervals along the width direction, as shown in Figure 2. The results show that the temperature distribution within the 30 mm gauge length is relatively uniform, with the maximum temperature at the center reaching approximately 480 °C and decreasing to about 450 °C at the edges of the gauge section. This results in a maximum temperature variation of approximately 30 °C.

**Figure 3:**
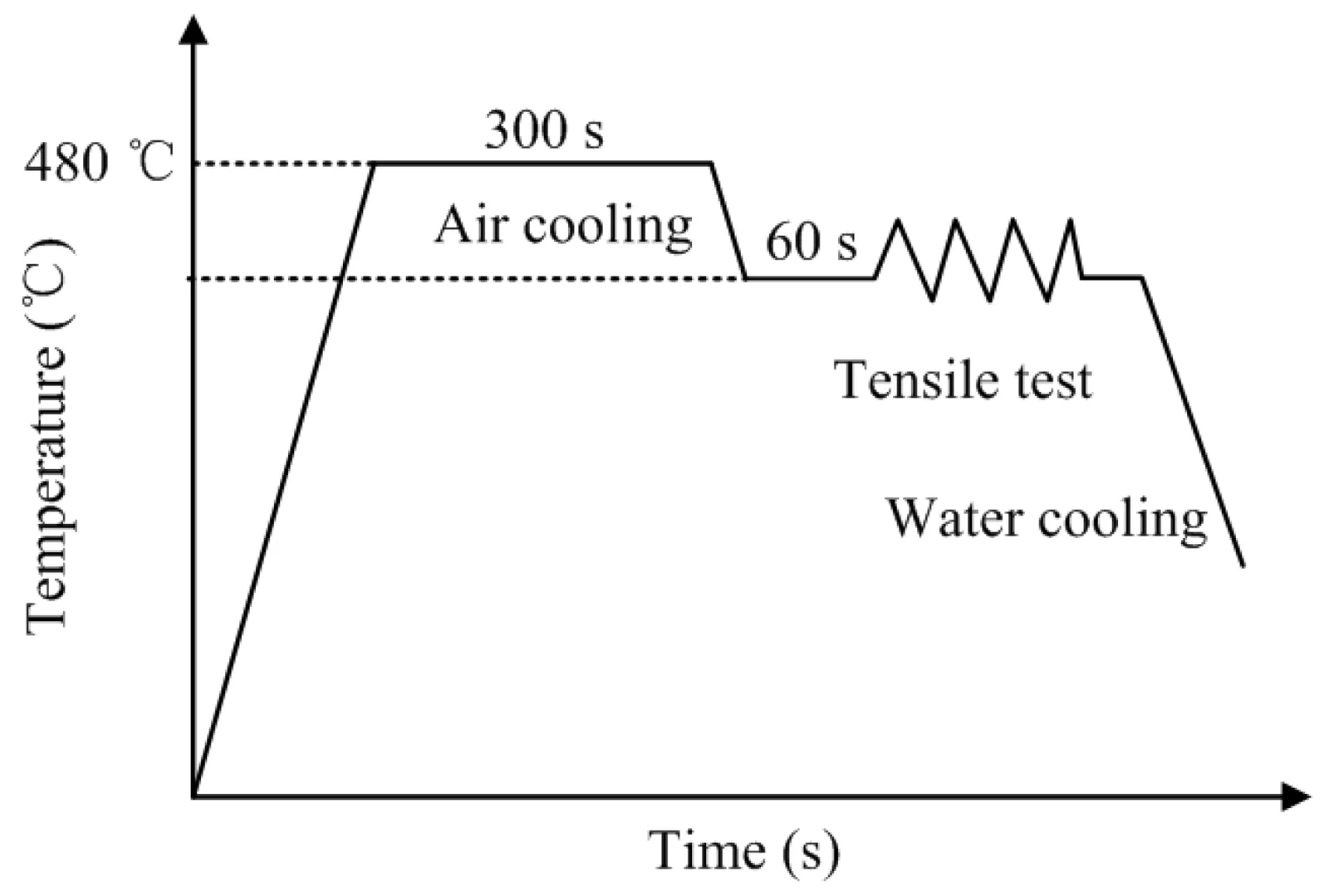
Procedure of the hot tensile test.

To simulate the hot stamping conditions in actual industrial production, a hot tensile test procedure was established, as shown in Figure 3. First, the specimen was heated to 480 °C and held for 300 s to ensure a complete solid solution. Following solution treatment, the precipitates dissolve, which reduces the resistance to dislocation motion, leading to a decrease in yield strength and an increase in ductility. Next, it was air-cooled to the target temperatures (350 °C, 400 °C, and 450 °C) and held for 60 s to achieve temperature uniformity. Subsequently, the specimen was tensile tested at target strain rates of 0.001, 0.01, and 0.1 s^−1^ until fracture. Each target condition was subjected to triplicate tensile tests, and the mean values were taken as the final experimental data.

#### 2.2.2. Hot Fracture Test

**Figure 4:**
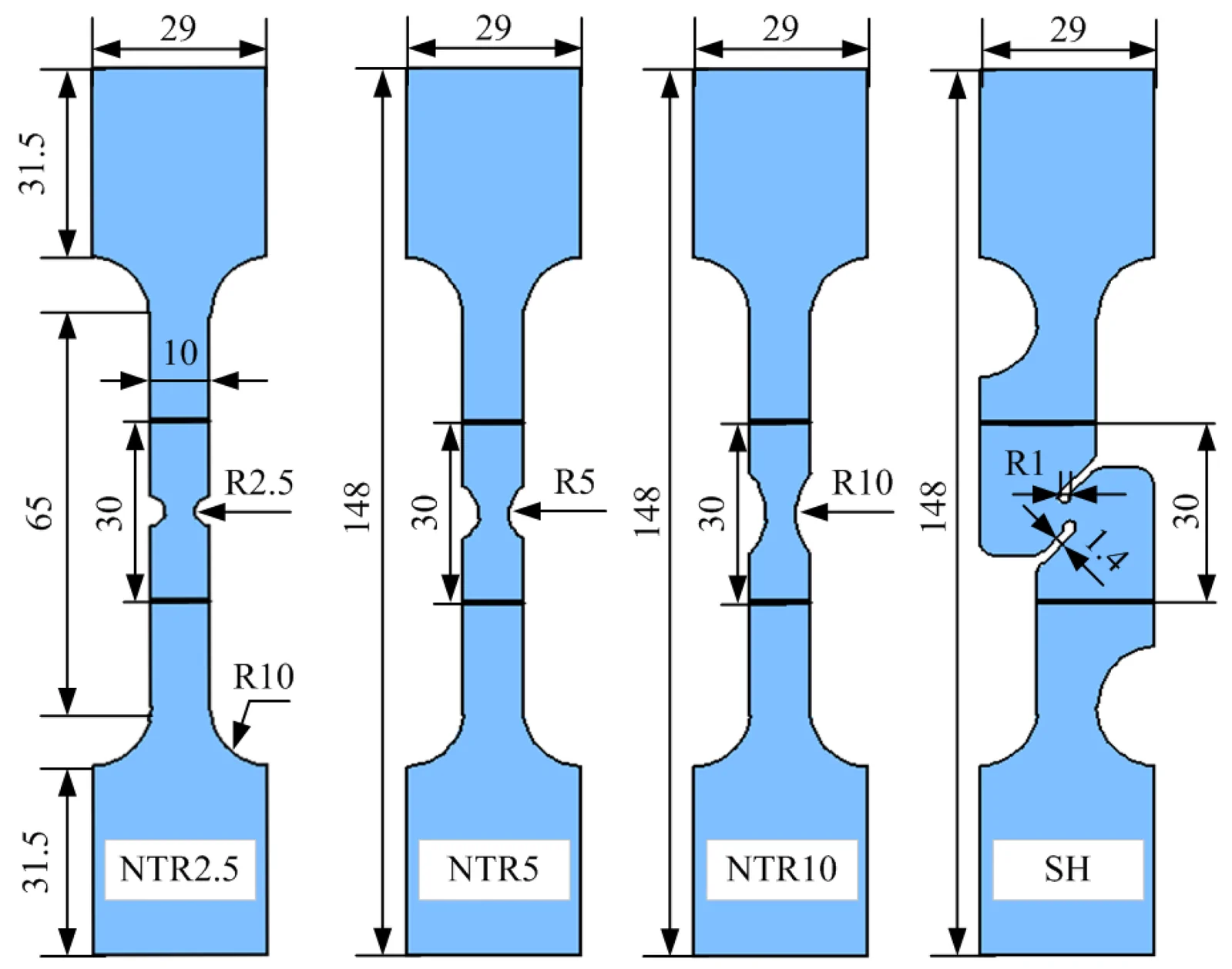
Shape and size of applied specimens (mm).

To investigate the deformation behavior and fracture response of 7075 aluminum alloy within the temperature ranges of 350–450 °C and the strain rate range of 0.001–0.1 s^−1^, NTR2.5, NTR5, NTR10 and SH were designed. The dimensions of each specimen are shown in Figure 4, and the initial gauge length of each specimen was set to 30 mm. All fracture tests were conducted on a 10 kN hydraulic universal testing machine equipped with an induction heating system. Before testing, high-temperature-resistant speckle patterns were prepared on the specimen surfaces, and the upper and lower boundaries of the gauge length were marked to facilitate subsequent extraction of the displacement via digital image correlation (DIC). During testing, two cameras (MER-030-120-GM/C-P, Daheng Imaging, Beijing, China) captured images at 5 fps, with an image resolution of 2048 × 2048 pixels, and uniform illumination was used to ensure sufficient image contrast. The DIC region of interest (ROI) was a rectangular area of 30 mm × 10 mm, covering the deformation region at the center of the specimen. In the recorded images, the corresponding ROI size was approximately 1500 × 500 pixels, and the average spatial resolution at the specimen center was approximately 0.02 mm/pixel. The system was calibrated using a standard calibration target before testing. After testing, the recorded speckle images were analyzed using Vic-3D 9, with a subset size of 17 pixels and a step size of 5 pixels.

The experimental design is shown in Table 2. To investigate the effect of temperature on fracture behavior, NTR2.5 specimens were used as reference specimens and tested at 350 °C, 400 °C, and 450 °C at a strain rate of 0.01 s^−1^. To investigate the effect of strain rate on fracture behavior, NTR2.5 specimens were tested at 0.001, 0.01, and 0.1 s^−1^ at a temperature of 350 °C. To investigate the effect of stress state on fracture behavior, NTR2.5, NTR5, NTR10, and SH specimens were tested at 350 °C and 0.01 s^−1^.

To ensure that each specimen was tested at the target strain rate, finite element simulations were performed for each specimen to determine the corresponding loading speed. Since 7075 aluminum alloy exhibits severe necking during hot tensile testing, the strain rate in the necking region continuously increases. Therefore, characterizing the strain rate of the specimen is a key issue. In this study, the moment corresponding to the peak point of the load–displacement curve was taken as the onset of necking, and the maximum strain rate in the finite element model at the onset of necking was defined as the reference strain rate. By applying different loading speeds to the finite element models, the loading speed at which the reference strain rate of each specimen reached the target value was determined as the experimental loading speed. The loading speeds for each specimen determined by finite element simulation are listed in Table 2.

**Figure 5:**
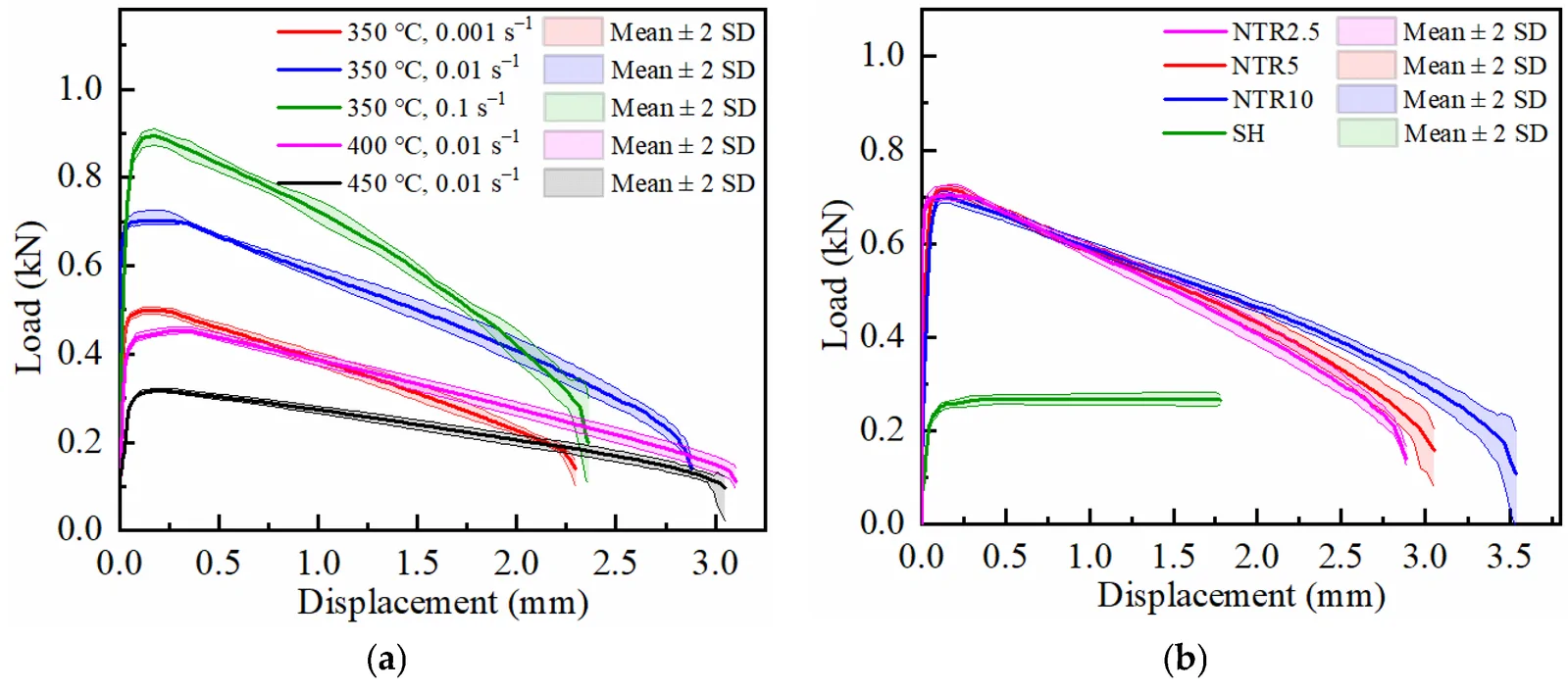
Representative load–displacement curves with mean ± 2 SD bands from three repeated tests for the fracture specimens: (**a**) NTR2.5 specimens; (**b**) 350 °C; 0.01 s^–1^.

To ensure the reliability of the experimental data and eliminate the influence of potential random defects in a single specimen, each target condition was tested in triplicate. The mean ± 2 standard deviation (Mean ± 2 SD) band of the three replicate tests is shown as the shaded region in Figure 5, which demonstrates excellent repeatability. Considering the physical correspondence between the load–displacement curves and the local strain field data recorded by DIC, and since the local strain field cannot be mathematically averaged, a representative specimen whose macroscopic mechanical response is closest to the mean was selected for subsequent analysis, as indicated by the solid line in Figure 5.

#### 2.2.3. Isothermal Bulging Test

**Figure 6:**
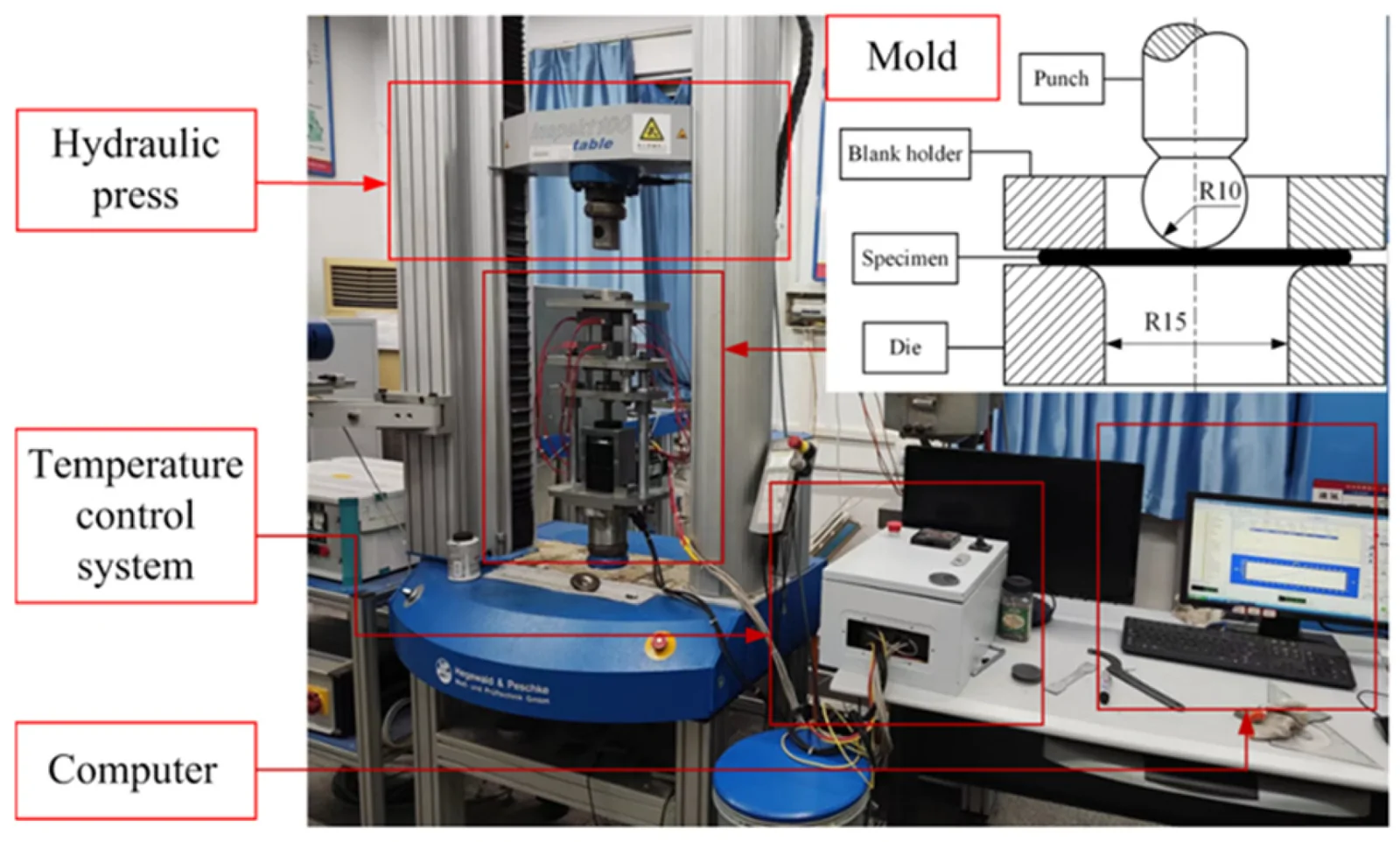
Schematic of the isothermal bulging test platform.

To verify the predictive capability of the established high-temperature semi-coupled damage model, an isothermal bulging test was conducted. The experimental equipment is shown in Figure 6 and consists of a universal testing machine, a bulging die, a temperature control system, and a computer control system. The bulging die comprises a die, a blank holder, and a punch. Heating rods are installed inside the die. The die temperature was measured by K-type thermocouples and fed back to the temperature control system to maintain the isothermal conditions required for bulging. Circular specimens with a diameter of 60 mm were machined from the original 7075 sheet and polished with sandpaper to ensure a smooth surface. Before testing, a graphite-based high-temperature lubricant was uniformly applied between the punch and the specimen. During testing, the specimen was first heated to 480 °C and held in a furnace for 300 s to ensure complete solution treatment. Then, the heat-treated specimen was rapidly transferred to a die preheated to 350 °C and held for 60 s to achieve temperature uniformity. Finally, the punch moved downward at a preset speed of 2.5 mm/s until the specimen fractured. The bulging tests were conducted in triplicate, and the mean values were adopted as the final experimental data.

## 3. Calibration Results

### 3.1. Calibration of the Viscoplastic Constitutive Model

**Figure 7:**
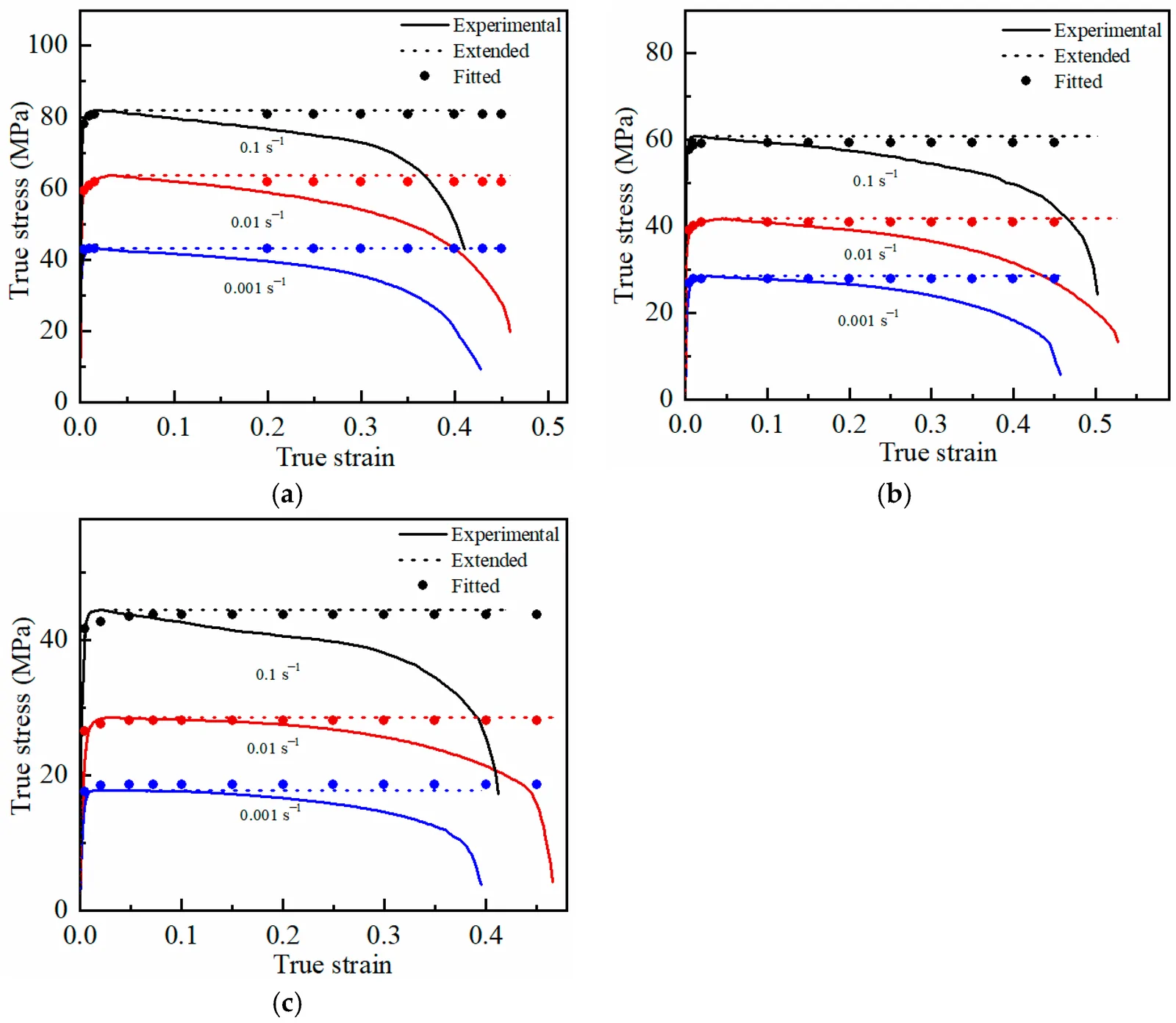
Extension and fitting of true stress–strain curves under different deformation conditions: (**a**) 350 °C; (**b**) 400 °C; (**c**) 450 °C.

The average load–displacement curves obtained from the hot tensile tests were converted into true stress–strain curves, as shown in Figure 7. Since the deformation after necking is no longer uniform and the stress state changes from uniaxial to triaxial, the post-necking data cannot represent the true mechanical behavior. To cover the plastic strain range required for subsequent constitutive model calibration, the true stress–strain curve before necking was extrapolated. The extrapolation begins at the peak stress point: the strain is extended to the target maximum value, while the stress is held constant at the peak value, as illustrated by the dashed line in Figure 7.

Based on the above extended true stress–strain data, the parameters in the visco-plastic constitutive model were calibrated using the optimization method proposed in Ref. [33]. In this study, the viscoplastic model was constructed in MATLAB 2018a using the forward Euler algorithm, and the optimization software Isight 5.9 combined with the non-dominated sorting genetic algorithm II (NSGA-II) was employed to identify a set of globally optimal parameters. The objective function was defined as the sum of squared logarithmic errors between the experimental and simulated stresses:

(13) $$f(x)=\frac{1}{M}\sum_{j=1}^{M}\left\{\frac{1}{N_{j}}\sum_{i=1}^{N_{j}}{\left(\ln\frac{\sigma_{ij}^{c}}{\sigma_{ij}^{e}}\right)}^{2}\right\}$$

where *f*(*x*) is the difference between the experimental stress and the model stress; *x* denotes the material parameters in the constitutive model; *N_j_* is the number of data points on the *j*-th stress–strain curve used in the calculation; $\sigma_{ij}^{e}$ is the experimental stress; $\sigma_{ij}^{c}$ is the calculated stress; and *M* is the number of curves.

Optimization was performed using the stress–strain curves at two test temperatures, with a total of six experimental curves. First, the temperature-independent parameters *n*_1_ and *n*_2_, as well as the values of *A*, *K*, *k*, *B*, *C*, and *E* at temperatures *T*_1_ (350 °C), and *T*_2_ (400 °C) were calibrated by the optimization algorithm. Then, by simultaneously solving the Arrhenius equations, *A*_0_, *K*_0_, *k*_0_, *B*_0_, *C*_0_, *E*_0_, and *Q_A_*, *Q_K_*, *Q_k_*, *Q_B_*, *Q_C_*, *Q_E_* were obtained. The parameter vector is *X* = [*A*^T1^, *K*^T1^, *k*^T1^, *B*^T1^, *C*^T1^, *E*^T1^, *n*_1_, *n*_2_, *A*^T2^, *K*^T2^, *k*^T2^, *B*^T2^, *C*^T2^, *E*^T2^]. The initial population space was set with a lower bound *X*_min_ = [0, 50, 1, 5, 200, 30,000, 0, 0, 0, 50, 0, 5, 300, 20,000] and an upper bound *X*_max_ = [100, 100, 15, 15, 300, 50,000, 10, 10, 50, 100, 5, 15, 400, 30,000]. The population size was set to 50, the number of generations to 100, the crossover rate to 0.8, and the mutation rate to 0.05. The convergence criterion was reaching the maximum number of generations. The calibration results for each parameter are listed in Table 3.

Based on the calibrated parameters, the stress–strain curves of 7075 aluminum alloy under different deformation conditions were obtained, as shown in Figure 7. The results demonstrate that the proposed constitutive model exhibits excellent predictive capability and accurately describes the hot deformation behavior of 7075 aluminum alloy.

### 3.2. Calibration of the Damage Initiation Model

To obtain the damage initiation strain, fracture strain, and stress state evolution of the four geometric specimens (NTR2.5, NTR5, NTR10, and SH) under the target conditions, numerical simulations of these specimens were performed using ABAQUS/Explicit. Considering that deformation is mainly concentrated within the gauge length while that outside is negligible, only the gauge length was modeled to improve computational efficiency. The boundary conditions were defined with one end fully fixed and the other end subjected to a displacement-controlled constraint. The material density and Poisson’s ratio were set to 2.81 × 10^−9^ t/mm^3^ and 0.33, respectively. The plastic behavior was governed by the Mises yield criterion, with the flow stress and hardening evolution described by the previously calibrated viscoplastic constitutive model, which was implemented into ABAQUS via a VUMAT user subroutine.

**Figure 8:**
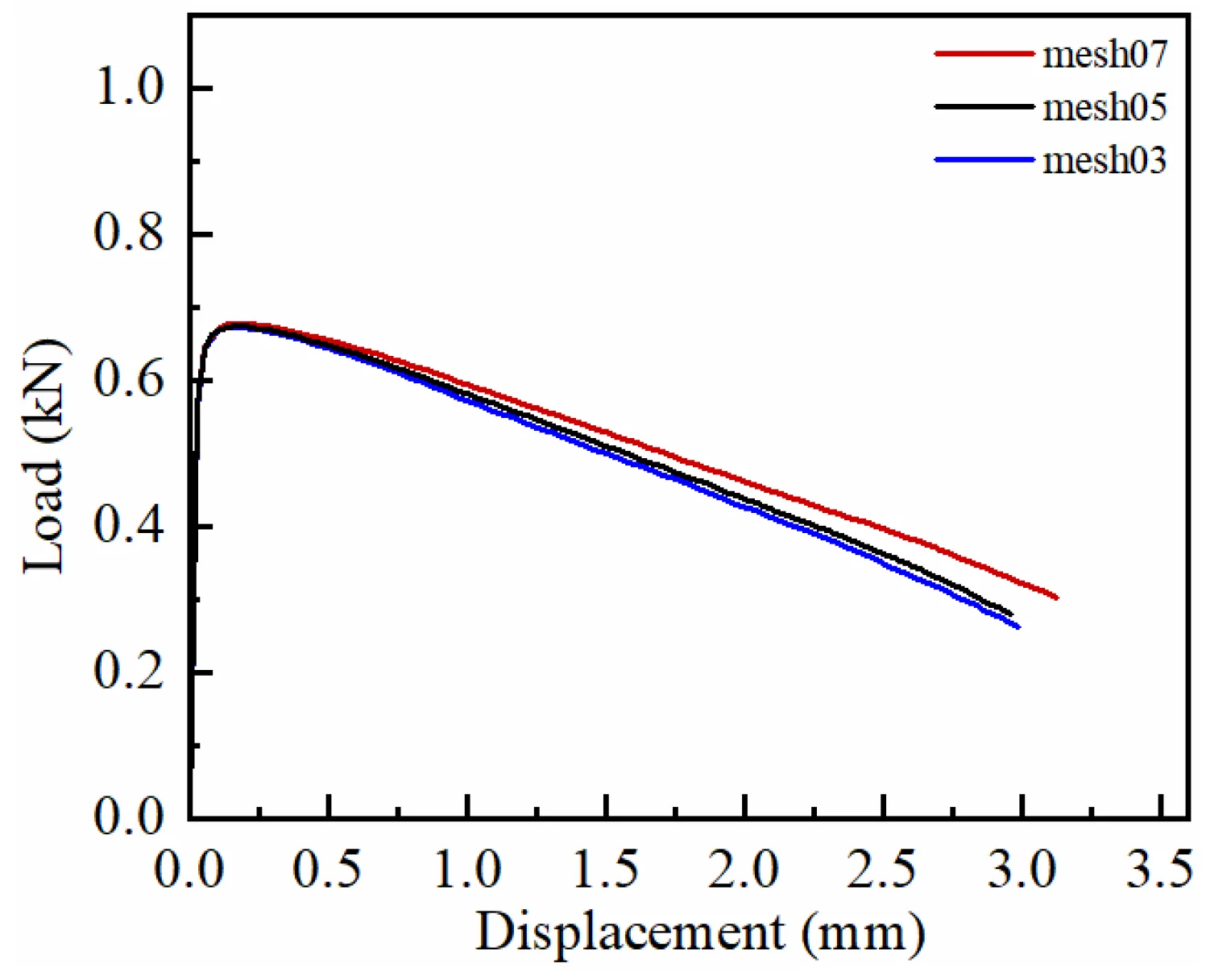
Comparison of simulated load–displacement curves for different mesh sizes.

To ensure mesh-independent results, a mesh sensitivity analysis was conducted on the NTR2.5 specimen. Three element sizes (0.7 mm, 0.5 mm, and 0.3 mm) were evaluated, and the comparison of load–displacement curves is presented in Figure 8. The results indicate that the coarse mesh (0.7 mm) overestimates the load-carrying capacity during the damage softening stage and exhibits delayed fracture. As the mesh is refined, the results converge; the curves obtained by the 0.5 mm and 0.3 mm meshes almost completely overlap, with a load–displacement RMSE of only 0.0097 kN and a relative error in fracture displacement of approximately 1%. Balancing computational efficiency and accuracy, a mesh size of 0.5 mm was applied in the deformation region of the NTR2.5, NTR5, and NTR10 specimens, and that in the remaining regions was set to 1 mm. For the SH specimen, which experiences more severe local deformation, a finer mesh size of 0.25 mm was applied in the deformation region, and that in the remaining regions was set to 0.5 mm. The models were meshed with 8-node linear brick elements with reduced integration (C3D8R), using four layers of elements in the thickness direction. Furthermore, since the severe deformation region of each specimen is localized within the central 15 mm, and the measured maximum temperature variation in this region is less than 10 °C, the temperature field within the gauge section was assumed to be uniform and set to the target temperature.

The parameters in the damage initiation model were calibrated based on the stress states under different loading paths and the corresponding damage initiation strain. Before parameter calibration, the damage initiation moment must be determined. In this study, the peak point of the load–displacement curve, i.e., the onset of necking, is approximated as the damage initiation moment. This is because the peak load corresponds to the end of uniform plastic deformation, after which necking begins. The stress state in the necking region changes from uniaxial to triaxial, and the local strain rate increases rapidly. These conditions all promote void nucleation and growth, and thus the peak load point can be reasonably regarded as the damage initiation moment. Meanwhile, direct experimental identification of damage initiation at high temperatures is very difficult and is coupled with temperature, strain rate, and microstructural evolution. In contrast, the peak load point is a well-defined and reproducible feature on the load–displacement curve, which facilitates parameter calibration. In addition, the semi-coupled fracture model focuses on the macroscopic mechanical property changes caused by damage evolution rather than the microscopic process of void evolution. Therefore, using the peak load point to determine the damage initiation moment is simple, effective, and reasonable. Based on the viscoplastic constitutive model, finite element simulations were performed on the SH, NTR2.5, NTR5, and NTR10 specimens at 350 °C and 0.01 s^−1^. The element with the maximum equivalent plastic strain at the damage initiation moment was defined as the critical element, and its equivalent plastic strain was taken as the damage initiation strain. The damage initiation strain for each specimen is listed in Table 4.

**Figure 9:**
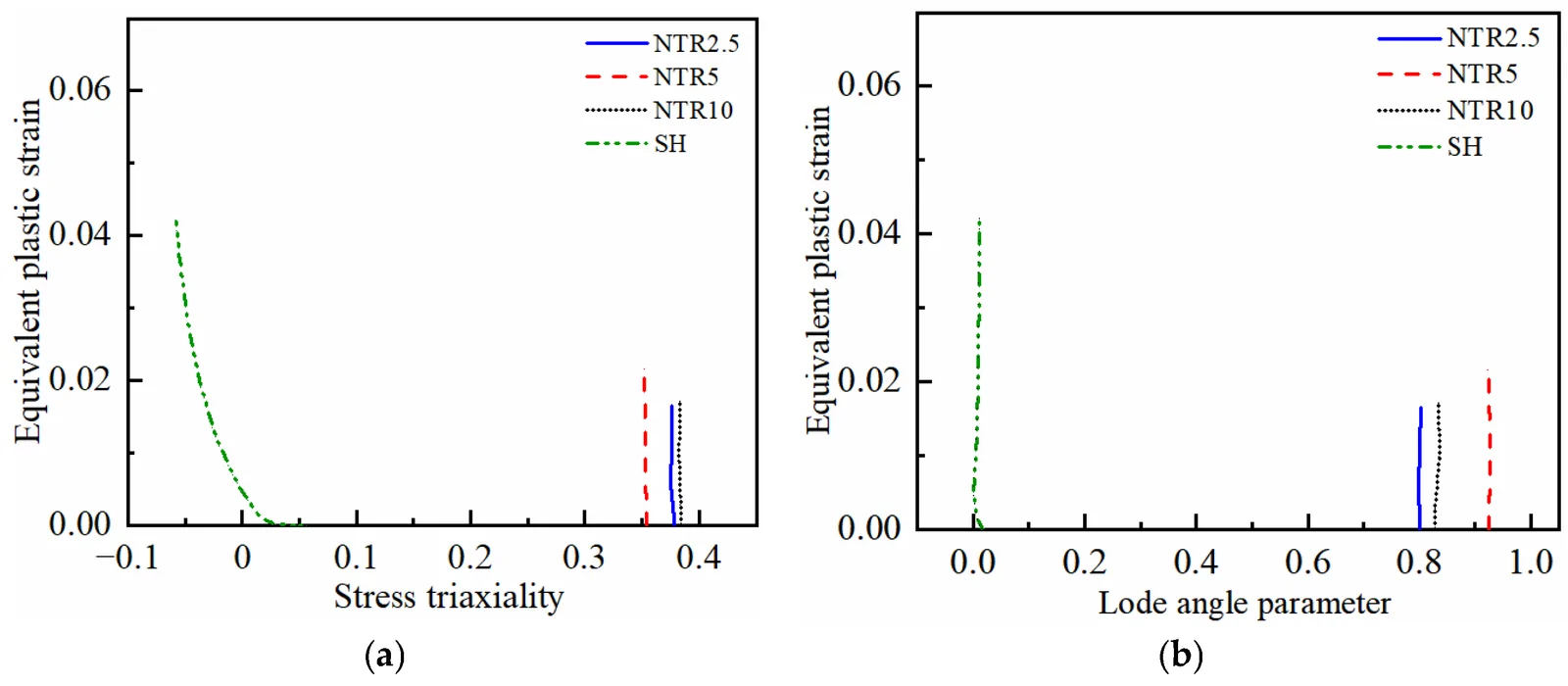
Stress state of each specimen during the damage initiation stage at 350 °C with 0.01 s^−1^: (**a**) Stress triaxiality; (**b**) Lode angle parameter.

The evolution of the stress state at the critical element during the damage initiation stage with equivalent plastic strain is shown in Figure 9. As can be seen from the figure, the stress triaxiality and Lode angle parameter of each specimen evolve approximately linearly during the damage initiation stage. Therefore, the stress state is characterized by the average stress triaxiality and the average Lode angle parameter. The parameters *b*_1_, *b*_2_, and *b*_3_ were determined using the NSGA-II optimization algorithm. The lower and upper bounds of *b*_1_, *b*_2_, and *b*_3_ were set to 0 and 20, respectively, and the initial value was set to 10. The objective function was defined as the error between the experimental and predicted damage initiation indicators. The population size was set to 50, the number of generations to 100, the crossover rate to 0.8, and the mutation rate to 0.05. The optimization was terminated upon reaching the maximum number of generations. The calibration results are listed in Table 5.

**Figure 10:**
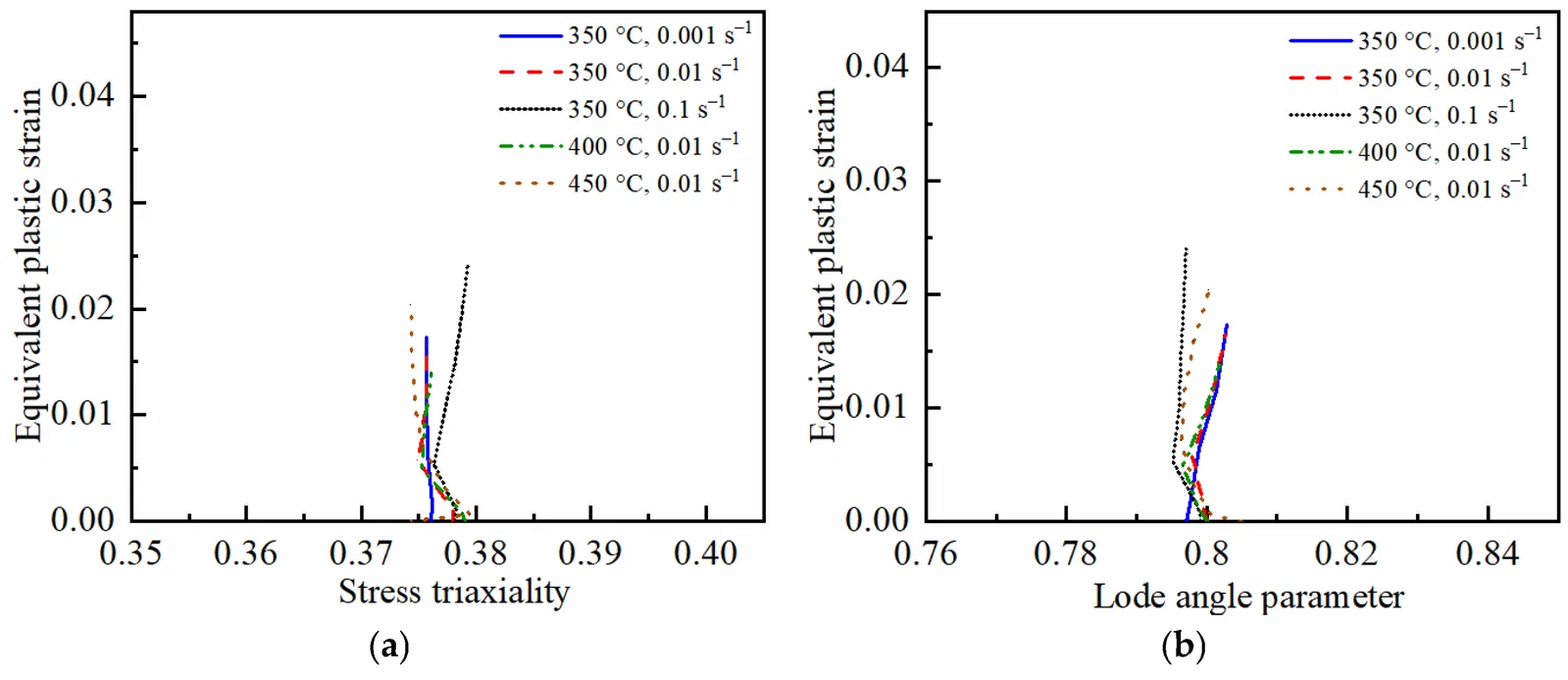
Stress state of NTR2.5 specimens during the damage initiation stage at different conditions: (**a**) Stress triaxiality; (**b**) Lode angle parameter.

Similarly, finite element simulations were performed on the NTR2.5 specimen under different deformation conditions. The extracted damage initiation strains are listed in Table 4, and the evolution of the stress state as a function of equivalent plastic strain is shown in Figure 10. While keeping parameters *b*_1_, *b*_2_, and *b*_3_ unchanged, the same NSGA-II algorithm was used to determine the values of parameters *F*_1_ and *F*_2_. The lower and upper bounds of *F*_1_ and *F*_2_ were also set to 0 and 20, respectively, with an initial value of 10. The objective function was the same as that used for calibrating *b*_1_–*b*_3_. The population size, number of generations, crossover rate, mutation rate, and convergence criterion were all the same as those described above. The calibration results are also listed in Table 5.

**Figure 11:**
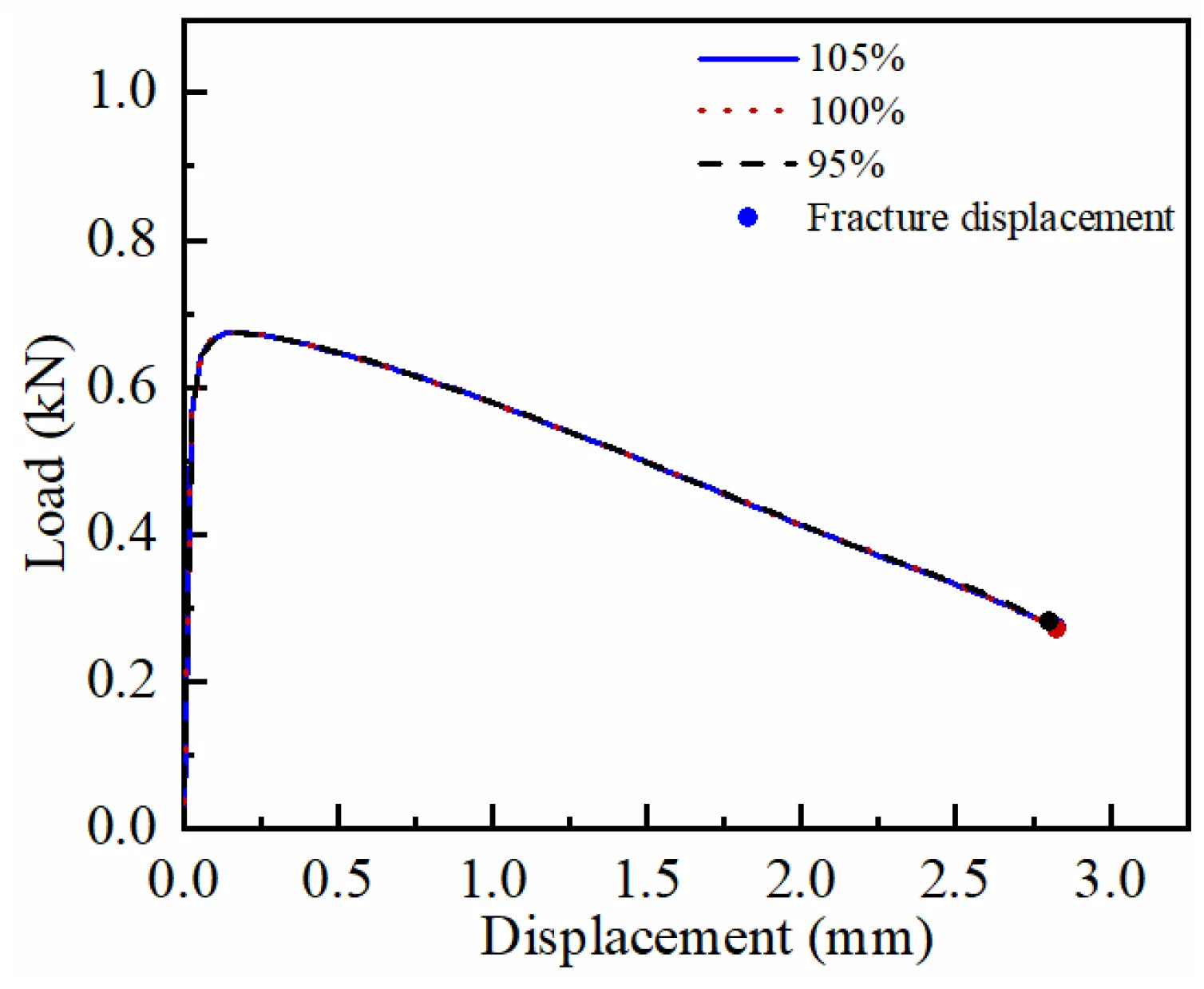
Comparison of simulation results under different damage initiation moment assumptions.

To further evaluate the influence of the damage initiation criterion assumption on the high-temperature semi-coupled fracture model, the damage initiation moment was set to 95%, 100%, and 105% of the onset of necking, respectively, and the damage initiation model parameters were recalibrated. Using the NTR2.5 specimen as a representative case, the simulation results under the three assumptions are compared in Figure 11. The results show that under these three assumptions, the predicted fracture displacement varies by less than 0.83%, and the RMSE of the load–displacement curve varies by less than 0.001 kN. This indicates that the shift in the damage initiation moment has little effect on the simulation results, and the model exhibits low sensitivity to this assumption.

### 3.3. Calibration of the Damage Evolution Model

**Figure 12:**
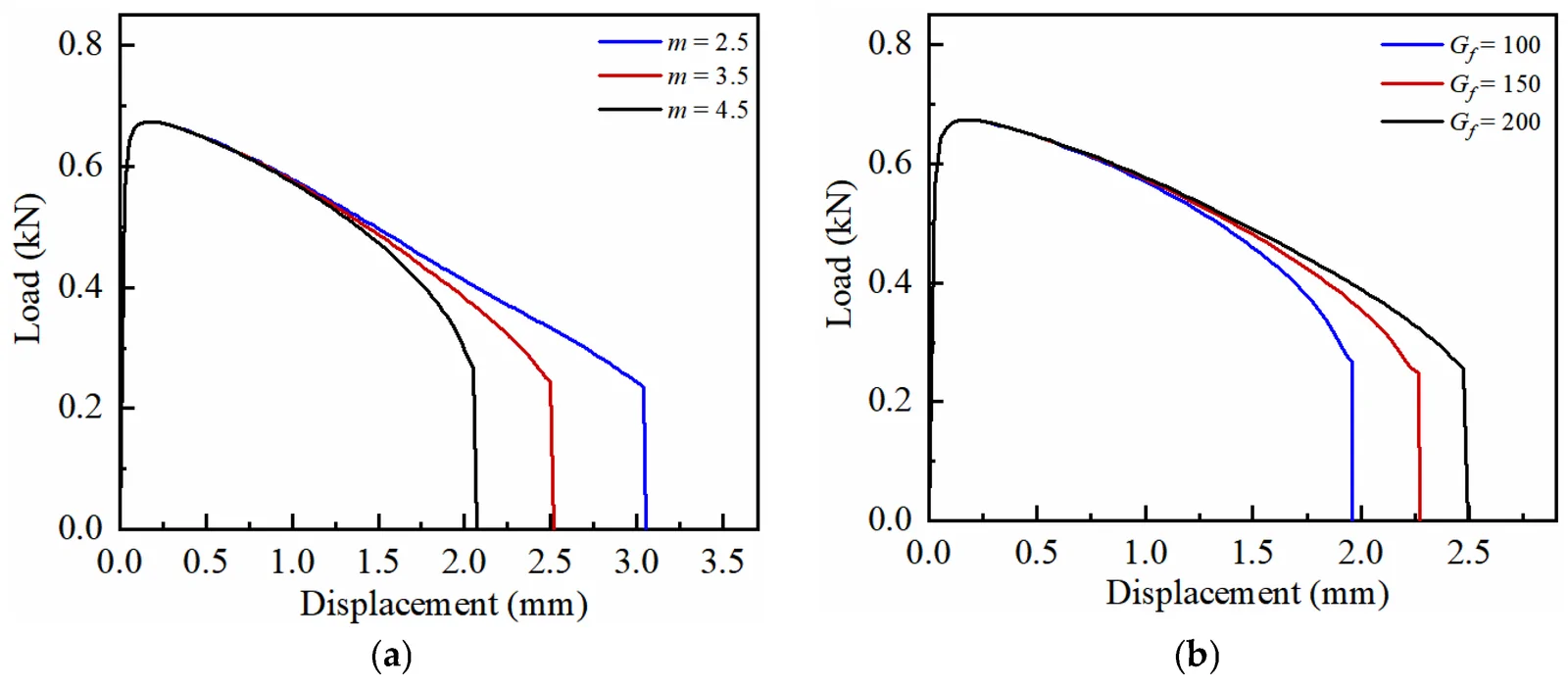
Local sensitivity analysis of the damage evolution parameters: (**a**) Effect of parameter *m* on the load–displacement response; (**b**) Effect of parameter $G_{f}$ on the load–displacement.

The parameters $G_{f}$ and *m* were calibrated using an inverse finite element method. By iteratively adjusting the parameter values, the simulated load–displacement curves gradually approach the experimental results. The parameter values should remain consistent for different specimens under the same deformation condition. To verify the identifiability of these two key parameters and rule out overfitting prior to finalizing the calibration, a local sensitivity analysis was conducted based on a representative condition using the NTR2.5 specimen at 350 °C and 0.01 s^−1^. First, $G_{f}$ was fixed at 150 mJ/mm^3^, while the parameter *m* was set to 2.5, 3.5, and 4.5, respectively. The extracted simulated load–displacement curves are shown in Figure 12a. The results indicate that varying *m* exclusively alters the post-peak softening slope, while the peak load remains unchanged. Subsequently, *m* was fixed at 4, and $G_{f}$ was set to 100, 150, and 200 mJ/mm^3^, as shown in Figure 12b. The results demonstrate that varying $G_{f}$ primarily shifts the final fracture displacement, while the preceding softening slopes remain parallel. Because *m* and $G_{f}$ govern distinct and independent geometric features of the load–displacement response, their cross-correlation is minimized. This confirms that the available experimental data are sufficient to uniquely determine these parameters.

Based on this verified calibration strategy, the parameter values at different temperatures and strain rates were determined and are listed in Table 6.

Table 6 indicates that the parameter *m* is temperature-dependent. The Arrhenius equation is used to describe the relationship between *m* and temperature *T*, and the fitting equation is given by:

(14) $$m=35.4558\exp\left(\frac{-12968.4734}{R_{g}T}\right)$$

The parameter $G_{f}$ is related to both temperature and strain rate. Trigonometric functions are introduced into the Arrhenius equation to describe the relationship among $G_{f}$, temperature, and strain rate. The fitting equation is given by:

(15) $$G_{f}=4.7292\exp(\frac{19128.7791}{R_{g}T})\cos\left[0.4279\ln(\frac{\dot{\bar{\varepsilon }}}{{\dot{\bar{\varepsilon }}}^{*}})\right]$$

It should be noted that the temperature-dependent exponential term and the strain-rate-dependent cosine term in Equation (15) are derived from physical mechanisms rather than arbitrary empirical fitting. The Arrhenius term reflects the thermal activation and ductile transition at high temperatures, leading to increased fracture energy. The cosine function is chosen not only to capture the non-linear rate sensitivity but also to ensure continuous smoothness, which is critical for preventing stress oscillation and ensuring numerical convergence in the ABAQUS explicit solver.

Once the damage initiation indicator reaches 1, the specimen begins to neck, and the local strain rate in the necking region continuously increases. Due to the relatively long necking stage during the high-temperature deformation of 7075 aluminum alloy, the nominal strain rate fails to represent the true local strain rate. Therefore, a strain rate correction is required. The evolution of the local strain rate as a function of equivalent plastic strain was extracted from the finite element simulation results, and the average strain rate ${\dot{\bar{\varepsilon }}}_{avg}$ was corrected as follows:

(16) $${\dot{\bar{\varepsilon }}}_{avg}=\frac{1}{\varepsilon^{p}}\int_{0}^{\varepsilon^{p}}\dot{\bar{\varepsilon }}(\varepsilon^{p})d\varepsilon^{p}$$

The corrected equation for $G_{f}$ is given by:

(17) $$G_{f}=4.7292\exp(\frac{19128.7791}{R_{g}T})\cos\left[0.3876\ln(\frac{\dot{\bar{\varepsilon }}}{{\dot{\bar{\varepsilon }}}^{*}})\right]$$

### 3.4. Calibration of the Fracture Model

**Figure 13:**
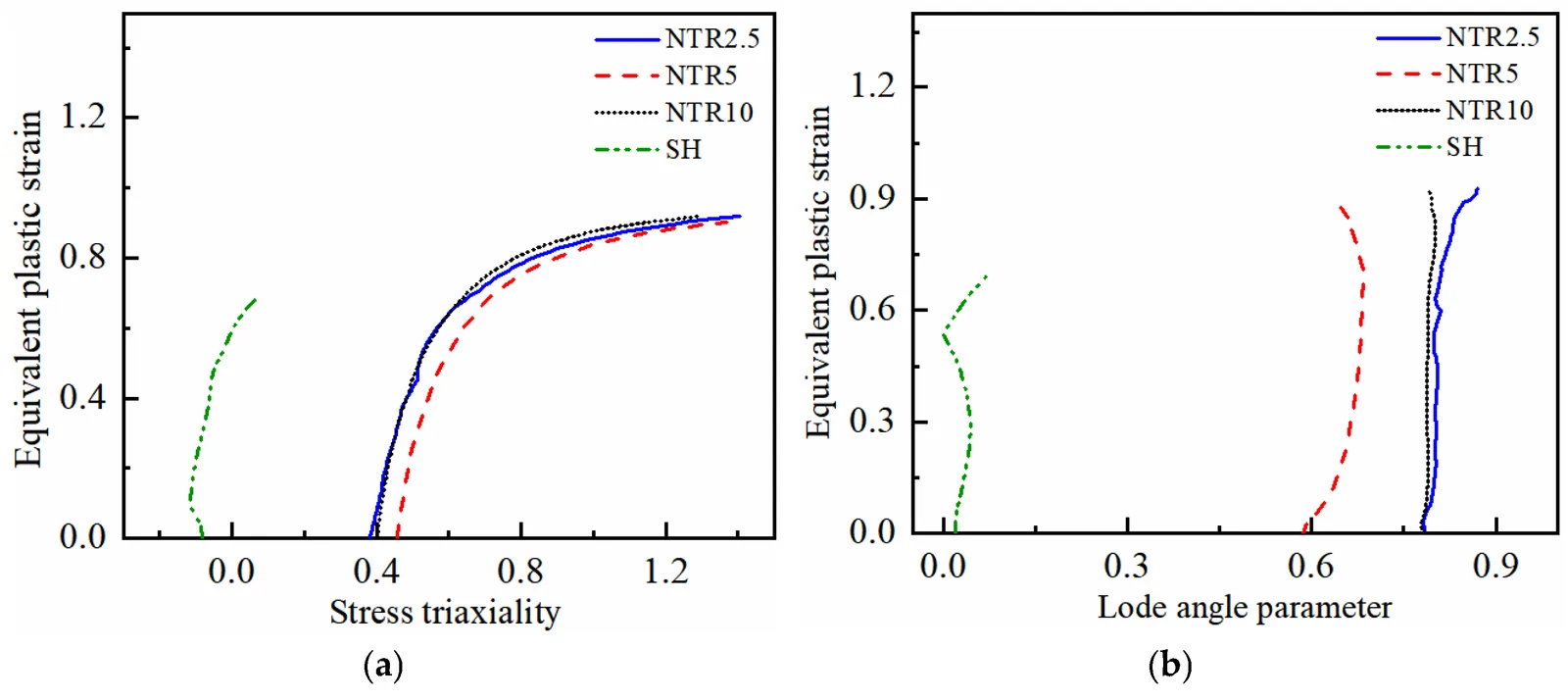
Stress state of each specimen during the damage evolution at 350 °C and 0.01 s^−1^: (**a**) Stress triaxiality; (**b**) Lode angle parameter.

**Figure 14:**
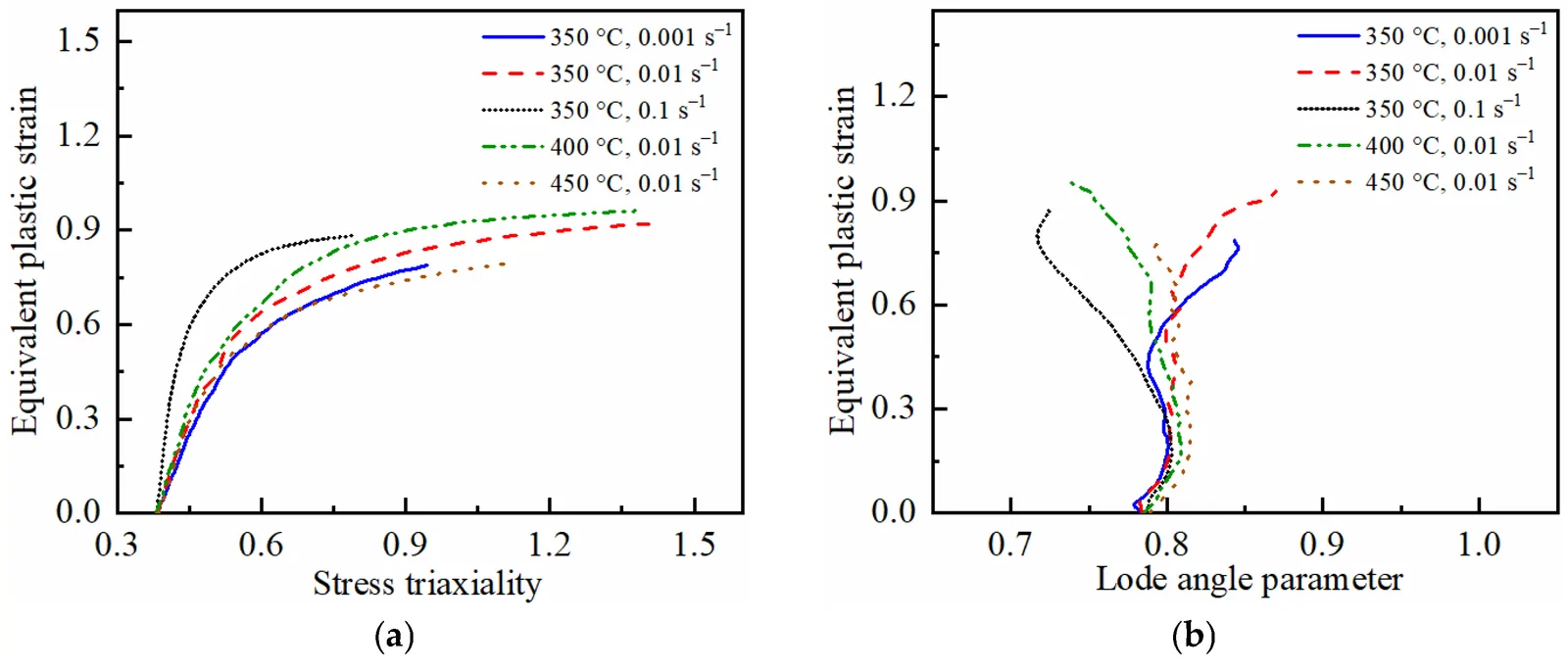
Stress state of NTR2.5 specimens during damage evolution at different conditions: (**a**) Stress triaxiality; (**b**) Lode angle parameter.

The fracture moment is defined as the point where the experimental load–displacement curve exhibits a sudden drop. The maximum equivalent plastic strain at this moment is extracted from the finite element simulation and defined as the fracture strain. The element exhibiting this maximum equivalent plastic strain is identified as the critical element. The evolution of its stress state from damage initiation to fracture is extracted, as shown in Figure 13 and Figure 14.

As can be seen from the figures, the stress triaxiality and lode angle parameter of the specimens vary significantly from damage initiation to fracture owing to the damage-induced necking phenomenon. Therefore, rather than using an average stress state, the fracture parameters were calibrated by evaluating the fracture indicator. Considering that the local strain rate continuously increases as necking becomes more severe, the strain rate evolution path was incorporated into the parameter calibration alongside the three-dimensional fracture locus. The optimization objective function is defined as:

(18) $$\left(c_{1},c_{2},c_{3},F_{3},F_{4}\right)=\left\{\left(c_{1},c_{2},c_{3},F_{3},F_{4}\right)|\sum_{i=1}^{N_{s}}{\left[I_{df}^{i}\left(c_{1},c_{2},c_{3},F_{3},F_{4}\right)-1\right]}^{2}\to \min\right\}$$

where *i* represents the *i*-th specimen; *N_s_* is the number of specimens used for calibration.

Similarly, the NSGA-II algorithm was employed to determine the parameter values. For *c*_1_, *c*_2_, *c*_3_, *F*_3_, and *F*_4_, the lower and upper bounds were set to [−5, −5, 0, −20, −5] and [5, 5, 10, 20, 5], respectively, with initial values of [0, 0, 5, 0, 0]. The objective function, population size, number of generations, crossover rate, mutation rate, and convergence criterion were identical to those described above. The calibrated parameters obtained from the optimization algorithm are listed in Table 7.

## 4. Discussion

### 4.1. Analysis of Hot Tensile Test

**Figure 15:**
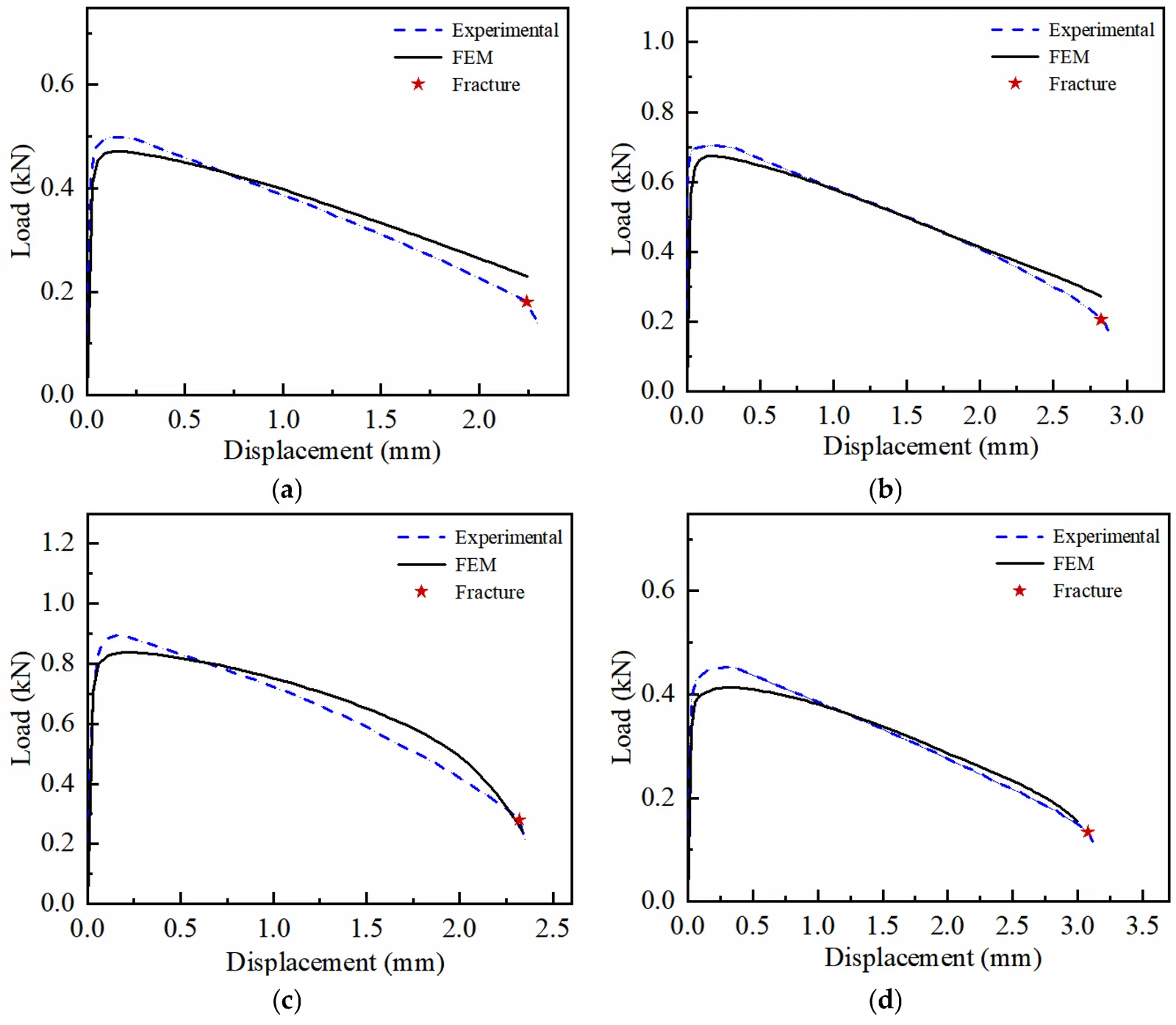

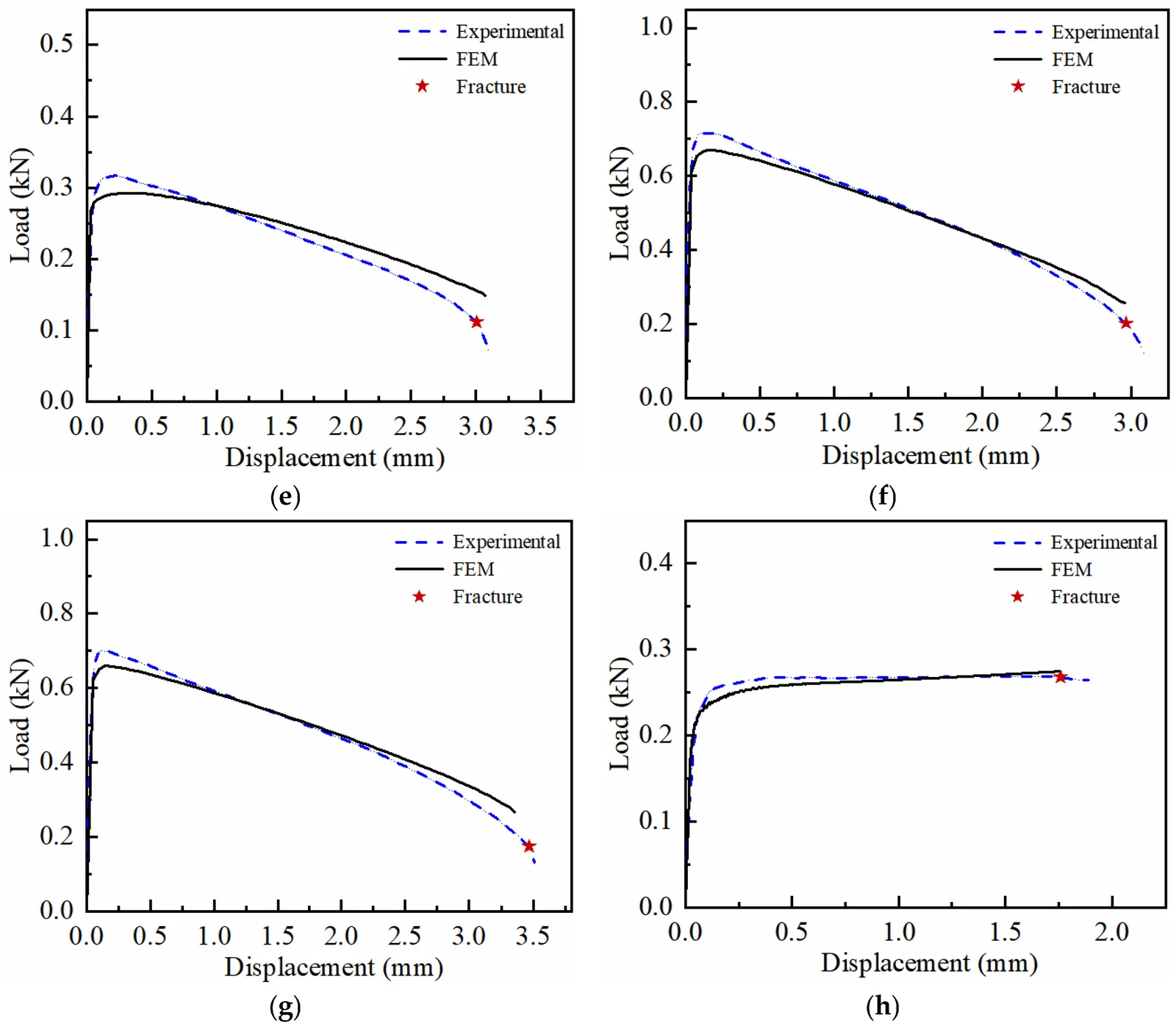
Comparison of load–displacement curves between simulation and experiment: (**a**) NTR2.5 (350 °C, 0.001 s^−1^); (**b**) NTR2.5 (350 °C, 0.01 s^−1^); (**c**) NTR2.5 (350 °C, 0.1 s^−1^); (**d**) NTR2.5 (400 °C, 0.01 s^−1^); (**e**) NTR2.5 (450 °C, 0.01 s^−1^); (**f**) NTR5 (350 °C, 0.01 s^−1^); (**g**) NTR10 (350 °C, 0.01 s^−1^); (**h**) SH (350 °C, 0.01 s^−1^).

Based on the established high-temperature semi-coupled damage model, the tensile process of each specimen was simulated using the finite element method (FEM). The simulated load–displacement curves were extracted and compared with the experimental results, as shown in Figure 15. It can be seen from the figure that at the initial yielding stage, the simulated load–displacement curves of each specimen are slightly lower than the experimental curves. This deviation is physically explained by the uniform temperature field assumption in the model. By assigning the central maximum temperature to the entire gauge section, the model ignores the strengthening effect of the cooler edges. In the damage evolution stage, the two sets of curves generally agree well, indicating that the model can accurately describe the damage-induced softening behavior of 7075 aluminum alloy during hot tensile testing.

For a quantitative assessment of the predictive accuracy, the correlation coefficient (*R*) and RMSE were selected as evaluation metrics. The calculated results under various deformation conditions are listed in Table 8. The results reveal a minimum *R* of 0.981 and a maximum RMSE of 0.047 kN, indicating that the model exhibits high predictive accuracy, accurately describing the hot deformation behavior of 7075 aluminum alloy.

Table 9 provides a direct comparison between the simulated and experimental fracture displacements. As shown in the table, the predicted values closely match the experimental results for all specimens, with a maximum relative error of 3.141%. This indicates that the model can accurately predict the fracture displacement of 7075 aluminum alloy during hot tensile testing.

**Figure 16:**
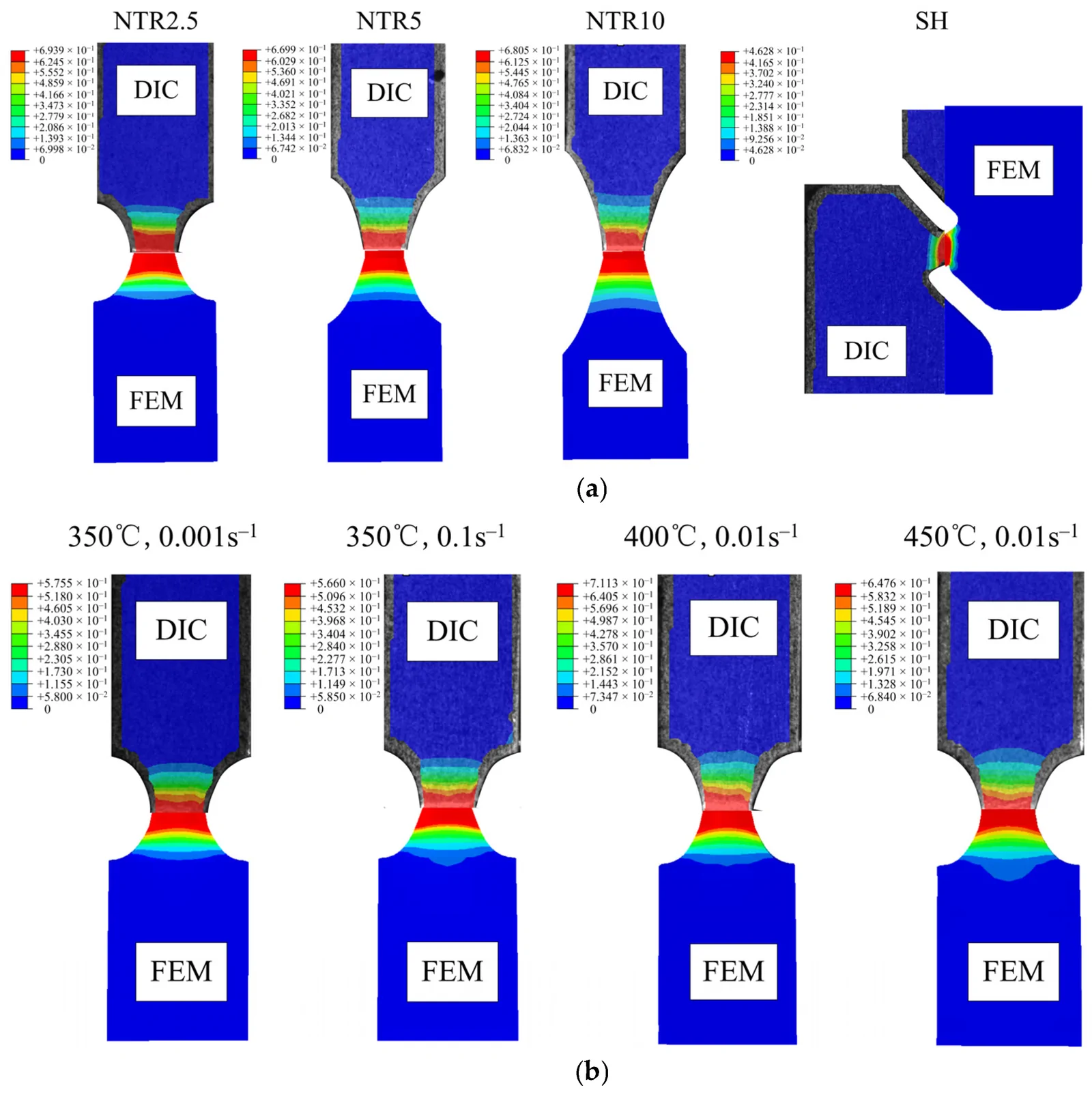
Comparison of strain field between simulation and experiment: (**a**) Strain fields of each specimen under the deformation condition of 350 °C and 0.01 s^−1^; (**b**) Strain fields of the NTR2.5 specimen under different deformation conditions.

The strain field distributions on the specimen surface at the fracture moment were obtained from the finite element simulation results and compared with the experimental strain fields measured by DIC, as shown in Figure 16. It can be seen from the figure that the two sets of results are generally consistent in terms of strain magnitude and distribution. The slight differences are attributed to the assumption of a uniform temperature field in the finite element model. In addition, the simulated fracture cross-sectional shapes for each specimen agree well with the experimental results. These results indicate that the model can effectively simulate the fracture behavior of 7075 aluminum alloy during hot tensile testing.

### 4.2. Analysis of Isothermal Bulging Test

Based on the established high-temperature semi-coupled damage model, an isothermal bulging finite element model was constructed in ABAQUS, and the forming process was numerically simulated. Due to symmetry, a half model was adopted and symmetric boundary conditions were applied. The die was defined as a rigid body and the blank as a deformable body. To determine the appropriate friction coefficient, multiple simulations were performed using different values. Previous studies have shown that the friction coefficient between the blank and the tool typically ranges from 0.05 to 0.3. It was found that when the friction coefficient was low, the maximum damage occurred at the top center of the specimen. As the friction coefficient increased, the location of maximum damage gradually shifted away from the center. When the friction coefficient was set to 0.225, the simulation results matched the experimental results most closely. Therefore, this value was adopted for the subsequent analyses. The blank holder force was set to 2.2 kN, and the downward speed of the punch was set to 2.5 mm/s. The blank adopted C3D8R elements with a mesh size of 0.5 mm × 0.5 mm, and four element layers were used through the thickness.

**Figure 17:**
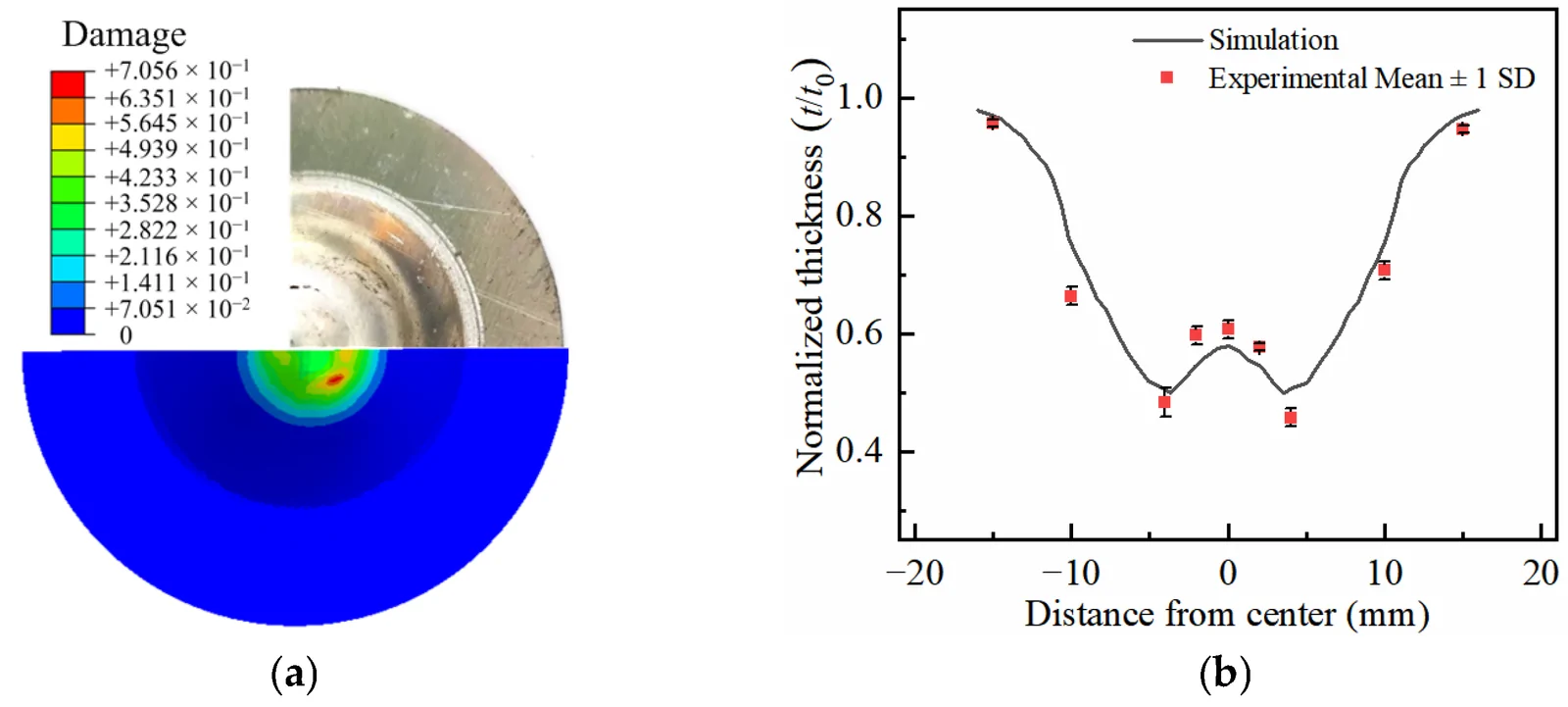
Comparison of fracture location and thicknesses in the experiment and simulation: (**a**) Fracture location; (**b**) Thicknesses.

The damage distribution of the blank at the fracture moment predicted by the finite element simulation is shown in Figure 17a. The damage at the center is 0.370, and it gradually increases with distance from the center, reaching a maximum of 0.706 at a radius of 4.203 mm, after which it begins to decrease. When the distance from the center exceeds 14.67 mm, the damage approaches zero because the local plastic strain is too small to reach the threshold for damage initiation. The experimental fracture location, taken as the average of three repeated tests, is approximately 4 ± 0.18 mm from the center, which is basically consistent with the location of maximum damage, with an average relative error of 5.10%. This indicates that the model can accurately predict the fracture location under isothermal bulging deformation.

Figure 17b compares the normalized thickness distributions obtained from the experiment and simulation. The experimental normalized thickness distribution is taken as the average of three repeated tests. The two distributions show a similar trend and correspond to the damage distribution, with the location of minimum thickness coinciding with that of maximum damage. To intuitively compare the thickness prediction accuracy, the relative errors between the experimental mean and the simulation at different positions were calculated, as listed in Table 10. It can be seen from Table 10 that the maximum relative error is 6.67%. The above results indicate that the model can accurately predict the fracture behavior of 7075 aluminum alloy at high temperatures.

### 4.3. Limitations and Future Work

Although the proposed high-temperature semi-coupled damage model exhibits high predictive accuracy for 7075 aluminum alloy, there are still some limitations that need to be addressed in future work. First, the model employs an isothermal assumption to improve computational efficiency, which neglects the local temperature gradient at the specimen edges and leads to a slight underestimation of the initial yield load. Second, to reduce the model’s complexity, an isotropic assumption is adopted, which ignores the potential anisotropy induced by texture evolution during hot deformation. Finally, the model has been strictly validated within the range of 350–450 °C and 0.001–0.1 s^−1^; however, extrapolating it to broader conditions requires further investigation.

In future work, a fully coupled thermo-mechanical framework and an anisotropic yield criterion will be introduced to further enhance the physical predictive capability of the model under complex loading conditions.

## 5. Conclusions

In this study, a high-temperature semi-coupled damage model was established within the framework of the semi-coupled fracture criterion. Based on this model, the fracture behavior of 7075 aluminum alloy during hot deformation was systematically investigated. The main conclusions are summarized as follows:

(1) A high-temperature semi-coupled fracture model incorporating the coupled effects of temperature, strain rate, and stress triaxiality was developed. It was found that the nonlinear damage evolution law more accurately captures the accelerated softening behavior at high temperatures compared to the traditional linear mode, providing a more physical representation of high-temperature deformation.

(2) The parameters of the high-temperature semi-coupled damage model were calibrated using an experimental–numerical hybrid method. To fully account for the local variation in strain rate caused by necking, the relationship between strain rate and equivalent plastic strain was introduced into the integral formula of the fracture indicator, thereby effectively correcting the strain rate.

(3) The proposed high-temperature semi-coupled damage model exhibits high predictive accuracy in hot tensile simulations. The simulated load–displacement responses, fracture displacements, and strain field distributions are highly consistent with the experimental results, yielding a maximum load–displacement RMSE of 0.047 kN and a maximum relative error of 3.141% in fracture displacement.

(4) The isothermal bulging tests provided independent validation for the established model. The results show that the average relative error for fracture location prediction is only 5.10%, and the maximum relative error for normalized thickness prediction is only 6.67%. These quantitative results fully confirm that the model can accurately capture the fracture behavior of 7075 aluminum alloy during hot deformation.

## Figures and Tables

**Table 1:** Experimental design and purposes.

Testing Types	Testing Condition	Purposes
Hot tensile test	Temperature: 350, 400, and 450 °C Strain rate: 0.001, 0.01, and 0.1 s^−1^	Calibrating the viscoplastic constitutive model
Hot fracture test	For NTR2.5: 350–450 °C, 0.001–0.1 s^−1^ For NTR5, NTR10, SH: 350 °C, 0.01 s^−1^	Calibrating the damage initiation, damage evolution, and fracture models
Isothermal bulging test	Temperature: 350 °C Speed: 2.5 mm/s	Validation

**Table 2:** Experimental design for fracture tests.

Specimen Types	Temperature (°C)	Strain Rate (s^−1^)	Loading Speed (mm/s)
NTR2.5	350	0.001	0.0056
		0.01	0.056
		0.1	0.56
	400	0.01	0.056
	450	0.01	0.056
NTR5	350	0.01	0.075
NTR10			0.1
SH			0.058

**Table 3:** Calibrated parameters of the viscoplastic constitutive model.

**Parameters**	**Results**	**Parameters**	**Results**	**Parameters**	**Results**
*K*_0_ (MPa)	3.2717	*B*_0_ (MPa)	0.1528	*A*_0_ (MPa)	2.0553 × 10^−10^
*Q*_K_ (J·mol^−1^)	1.7592 × 10^4^	*Q*_B_ (J·mol^−1^)	2.2869 × 10^4^	*Q*_A_ (J·mol^−1^)	1.3713 × 10^5^
*k*_0_ (MPa)	1.5323 × 10^−9^	*C*_0_ (MPa)	3.1524 × 10^3^	*E*_0_ (MPa)	22.2232
*Q*_k_ (J·mol^−1^)	1.1807 × 10^5^	*Q_C_* (J·mol^−1^)	1.2414 × 10^4^	*Q_E_* (J·mol^−1^)	3.9548 × 10^4^
*n*_1_	5.13	*n*_2_	5.41		

**Table 4:** The damage initiation strain for each specimen.

Specimen Types	Temperature (°C)	Strain Rate (s^−1^)	${\bar{\varepsilon }}_{ddi}^{p}$
NTR2.5	350	0.001	0.0173
		0.01	0.0166
		0.1	0.0241
	400	0.01	0.0139
	450	0.01	0.0204
NTR5	350	0.01	0.0216
NTR10			0.0172
SH			0.0421

**Table 5:** Calibration parameters of the damage initiation model.

*b*_1_	*b*_2_	*b*_3_	*F*_1_	*F*_2_
8.6665	4.2948	0.0625	0.07816	2.3891

**Table 6:** Inverse results of damage evolution model.

Specimen Types	Temperature (°C)	Strain Rate (s^−1^)	$G_{f}$ (mJ/mm^3^)	*m*
NTR2.5	350	0.001	105	2.9
		0.01	190	2.9
		0.1	113	2.9
	400	0.01	144	3.4
	450	0.01	118	4.1
NTR5	350	0.01	190	2.9
NTR10			190	2.9
SH			190	2.9

**Table 7:** Calibration parameters of the fracture model.

*c*_1_	*c*_2_	*c*_3_	*F*_3_	*F*_4_
−0.5032	−0.3126	0.7957	0.0433	−0.2966

**Table 8:** The *R* and RMSE of the predicted load–displacement results for each specimen.

Specimen Types	Temperature (°C)	Strain Rate (s^−1^)	*R*	RMSE (kN)
NTR2.5	350	0.001	0.990	0.024
		0.01	0.996	0.022
		0.1	0.984	0.047
	400	0.01	0.992	0.018
	450	0.01	0.981	0.019
NTR5	350	0.01	0.997	0.022
NTR10			0.995	0.024
SH			0.996	0.007

**Table 9:** Comparison of simulated and experimental fracture displacements.

Specimen Types	Temperature (°C)	Strain Rate (s^−1^)	Experimental (mm)	Predicted (mm)	Relative Error (%)
NTR2.5	350	0.001	2.241	2.246	0.223
		0.01	2.826	2.825	0.354
		0.1	2.320	2.337	0.733
	400	0.01	3.078	3.014	2.079
	450	0.01	3.006	3.076	2.329
NTR5	350	0.01	2.969	2.962	0.236
NTR10			3.470	3.361	3.141
SH			1.759	1.756	0.171

**Table 10:** Relative error of the normalized thickness between experiment and simulation.

Location (mm)	Experimental	Simulation	Relative Error (%)
15	0.95	0.97	2.11
10	0.73	0.75	2.74
4	0.48	0.49	2.08
2	0.57	0.55	3.51
0	0.60	0.56	6.67
−2	0.58	0.55	5.17
−4	0.48	0.50	4.17
−10	0.69	0.73	5.80
−15	0.96	0.97	1.04

## Data Availability

The original contributions presented in this study are included in the article. Further inquiries can be directed to the corresponding author.
